# Identifying genetically driven clinical phenotypes using linear mixed models

**DOI:** 10.1038/ncomms11433

**Published:** 2016-04-25

**Authors:** Jonathan D. Mosley, John S. Witte, Emma K. Larkin, Lisa Bastarache, Christian M. Shaffer, Jason H. Karnes, C. Michael Stein, Elizabeth Phillips, Scott J. Hebbring, Murray H. Brilliant, John Mayer, Zhan Ye, Dan M. Roden, Joshua C. Denny

**Affiliations:** 1 Department of Medicine, Vanderbilt University, Nashville, Tennessee 37232, USA; 2 Department of Epidemiology and Biostatistics, University of California, San Francisco, California 94158, USA; 3 Biomedical Informatics, Vanderbilt University, Nashville, Tennessee 37203, USA; 4 Center for Human Genetics, Marshfield Clinic Research Foundation, Marshfield, Wisconsin 54449, USA; 5 Biomedical Informatics Research Center, Marshfield Clinic Research Foundation, Marshfield, Wisconsin 54449, USA

## Abstract

We hypothesized that generalized linear mixed models (GLMMs), which estimate the additive genetic variance underlying phenotype variability, would facilitate rapid characterization of clinical phenotypes from an electronic health record. We evaluated 1,288 phenotypes in 29,349 subjects of European ancestry with single-nucleotide polymorphism (SNP) genotyping on the Illumina Exome Beadchip. We show that genetic liability estimates are primarily driven by SNPs identified by prior genome-wide association studies and SNPs within the human leukocyte antigen (HLA) region. We identify 44 (false discovery rate *q*<0.05) phenotypes associated with HLA SNP variation and show that hypothyroidism is genetically correlated with Type I diabetes (rG=0.31, s.e. 0.12, *P*=0.003). We also report novel SNP associations for hypothyroidism near HLA-DQA1/HLA-DQB1 at rs6906021 (combined odds ratio (OR)=1.2 (95% confidence interval (CI): 1.1–1.2), *P*=9.8 × 10^−11^) and for polymyalgia rheumatica near C6orf10 at rs6910071 (OR=1.5 (95% CI: 1.3–1.6), *P*=1.3 × 10^−10^). Phenome-wide application of GLMMs identifies phenotypes with important genetic drivers, and focusing on these phenotypes can identify novel genetic associations.

## Abstract (web summary)

Use of general linear mixed models (GLMMs) in genetic variance analysis can quantify the relative contribution of additive effects from genetic variation on a given trait. Here, Jonathan Mosley and colleagues apply GLMM in a phenome-wide analysis and show that genetic variations in the HLA region are associated with 44 phenotypes, 5 phenotypes which were not previously reported in GWASes.

## Graphical abstract

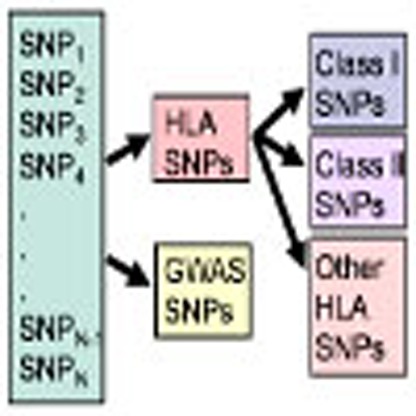

Electronic health records (EHRs) linked to genetic data have become an efficient tool to identify genetic factors modulating clinical phenotypes^1^,^2^,^3^,^4^,^5^. The integration of large amounts of individual-level genetic and phenotypic data provides a unique opportunity to identify genetically mediated disease entities and have enabled hypothesis-free approaches such as phenome-wide association studies (PheWAS)^6^,^7^,^8^,^9^,^10^. While a broad range of phenotypes can be rapidly extracted from EHR data sources, only a portion of these phenotypes may be modulated by underlying genetics. Indeed, many diseases currently lack clinical or genetic heritability (or liability) estimates. We hypothesized that linear mixed models could be used to rapidly identify and characterize genetically modulated phenotypes among a large set of EHR-derived phenotypes. These genetic phenotypes could then be subjected to more detailed genetic analysis.

Variance component approaches that employ generalized linear mixed models (GLMMs) quantify the additive phenotypic genetic variation attributable to a collection of common single-nucleotide polymorphisms (SNPs) genotyped in unrelated individuals^11^,^12^,^13^. For continuous traits, this variation represents a narrow-sense (additive) estimate of the phenotypic variability attributable to the SNPs. For binary traits, such as those used in these analyses, the models estimate the proportion of genetic variation captured on a continuous underlying liability scale so that estimates are not biased by a disease's prevalence^14^. The estimated genetic variation often exceeds the variance attributable to SNPs significantly associated by genome-wide association studies (GWAS)^15^,^16^. Hence, these methods are sensitive for ascertaining whether additive genetics modulate phenotypic risk^15^,^17^. These approaches can also parse the genetic variance across subsets of SNPs, which can facilitate the biological interpretation of sources of this variability^18^. These features of GLMMs make this approach well-suited to identifying the genetically driven phenotypes among a large collection of phenotypes.

We evaluate our hypothesis using a mixed-modelling approach in conjunction with 1,288 binary phenotypes defined by EHR-based PheWAS phenotypes^6^,^19^, which are derived from International Classification of Disease, Ninth revision, Clinical Modification (ICD-9)^20^ billing codes, ascertained in 29,349 subjects of European ancestry (EA) who were genotyped on the Illumina Exome Beadchip. Of relevance to these analyses, the exome chip contains limited and highly selective common SNP variation, including an over-representation of SNPs with phenotypic associations identified by GWAS and dense coverage of common SNPs in the human leukocyte antigen (HLA) region on chromosome 6p. We use GLMMs to show that many phenotypes have an underlying genetic risk attributable to SNPs on the exome chip, and that this risk is almost entirely attributable to the HLA-associated or GWAS-associated SNPs. We then identify specific SNPs associated with two HLA-associated phenotypes using GWAS, and replicate these findings. Our results support the use of linear mixed modelling to identify genetic phenotypes and demonstrate how this approach can efficiently lead to discovery of new genetic loci associated with phenotypes.

## Results

### SNP variation in HLA underlies many liability estimates

**Figure 1:**
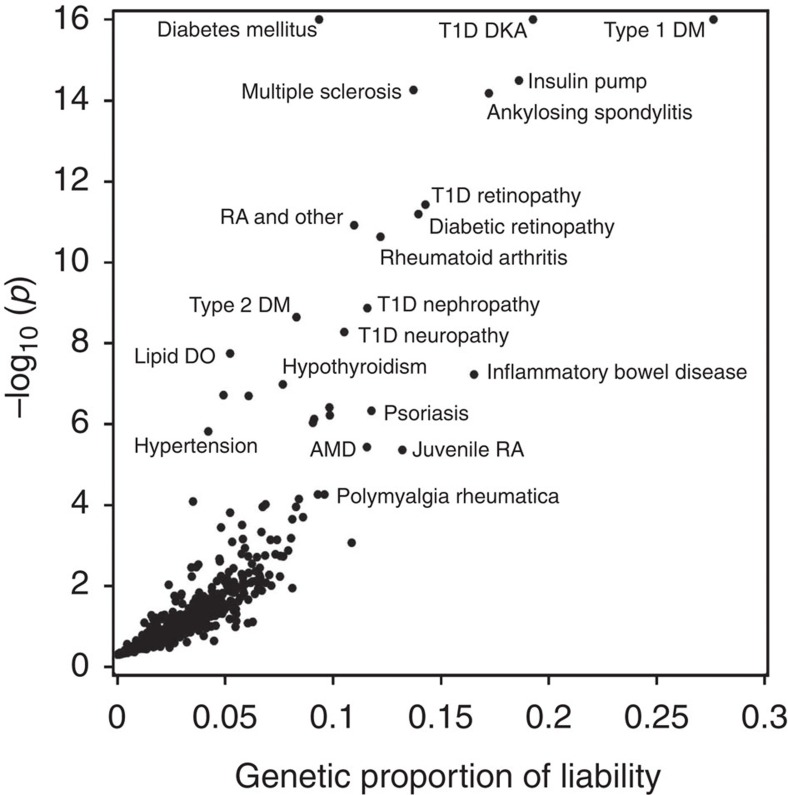
Genetic liability estimates and *P*-values for PheWAS phenotypes. Each point represents the results from a mixed-models analysis using all SNPs with MAF>0.01 on the exome chip, adjusted for age, sex and 20 principal components. PheWAS phenotypes with *P*-values<10^−16^ (3 phenotypes) were set to 10^−16^ for display purposes. Phenotypes with a genetic liability estimate<0 are not shown.

The analyses used 29,349 subjects of EA with existing exome array data extracted from BioVU, Vanderbilt's EHR-linked DNA biobank. Approximately 13% of subjects were under 18 years old and 53% were men (Supplementary Table 1). Subjects had a median length of follow-up of 8 years (interquartile range (IQR) 4–14) and a median number of 22 (IQR 10–41) distinct PheWAS-based phenotypes—typically diseases or other clinical findings. A list of the most common phenotypes is shown in Supplementary Table 1. We determined the genetic liability attributable to all common autosomal SNPs (minor allele frequency (MAF)>1%) on the exome chip (*n*=33,233 SNPs) for 1,288 PheWAS-defined clinical phenotypes with 50 or more cases (Fig. 1 and Supplementary Data 1). A quantile plot shows a higher-than-expected rate of low *P*-values, indicating that liability estimates for some phenotypes are greater than expected by chance (Supplementary Fig. 1a). To ensure unbiased liability estimates, the Restricted Maximum Likelihood (REML) statistical model was not constrained to force heritability estimates to be positive. Hence, 461 (35.8%) phenotypes had a negative heritability estimate. While a negative value does not have a biological interpretation, it suggests that the genetic liability is likely small. Phenotypes with negative heritability estimates tended to have smaller numbers of cases (median cases=211, IQR=102–467) than phenotypes with positive estimates (median=385, IQR: 153–1,001), suggesting that model instability was due to small numbers of cases. Of those phenotypes with positive heritability estimates, 68 were significant at a false discovery rate (FDR) threshold of 0.1, and 56 were significant at FDR *q*<0.05. When we restricted the analyses to phenotypes with over 200 cases, there were 58 and 46 phenotypes, respectively, that were significant at these thresholds. Two phenotypes with a negative heritability estimate had an FDR *q*<0.1, and both had fewer than 100 cases.

Two significant sources of biologically significant common SNP variation captured on this array are SNPs with significant associations (*P*<5 × 10^−8^) reported in the NHGRI GWAS Catalog (*n*=2,199, 6.6% of SNPs) and SNPs located around the immune-associated HLA gene cluster of chromosome 6p (*n*=2,147, 6.3%). When liability estimates were calculated omitting SNPs in the HLA region, there was a marked decrease in the number of phenotypes with significant liability estimates (Supplementary Fig. 1b), indicating that SNP variation in this region was a significant contributor to genetic liability estimates for many of the most significant phenotypes. When we further removed SNPs with phenotype associations reported in the GWAS Catalog (4,641 SNPs, including SNPs in linkage disequilibrium with the Catalog SNPs (*r*^2^>0.2)), the distribution of *P*-values approached expectations based on chance (Supplementary Fig. 1c). Hence, genetic variation seen using the exome chip was driven primarily by HLA SNPs and SNPs previously identified by GWAS. Based on these analyses, further exploration of phenotypes associated with SNP variation in the HLA region was expected to offer the most potential for new discovery.

### Analysis of phenotypes associated with HLA SNP variation

We identified phenotypes that had a significant genetic liability associated with the SNPs located in the HLA region to more fully elucidate the spectrum of HLA-associated diseases. HLA-associated phenotypes were identified by modelling two variance components (using two genetic relationship matrices) in the mixed model, one comprising SNPs located around the HLA region and the other comprising the remaining SNPs, and testing whether the liability associated with HLA component was significantly greater than 0. Many phenotypes were associated with SNP variability in the HLA region (Supplementary Data 2). In order to select a *P*-value threshold where there was optimal enrichment in phenotypes likely to represent true associations, we tallied the number of clinically recognized auto-immune diseases for three *P*-value bins. At FDR *q*<0.05, there was the most marked enrichment of auto-immune diseases (26 of 44 phenotypes below this threshold) (Supplementary Figs 1d and 2). Among the 18 other phenotypes below this threshold, there were a number of diagnoses that could have an immune-mediated aetiology but were not specific, such as ‘Other demyelinating diseases of the central nervous system', which describes the pathological findings associated with multiple sclerosis. Thirteen of the 44 phenotypes were represented in the GWAS Catalog (Table 1). Of the remaining phenotypes, only five corresponded to specific disease entities. Four of the five (sicca syndrome, dermatomyositis/polymyositis, polymyalgia rheumatic, cholangitis) are known autoimmune diseases^21^, while the fourth, dermatophytosis, has variably been associated with the HLA region^22^,^23^.

Genes within the HLA region activate immune responses through different mechanisms and are grouped into two major histocompatibility complex (MHC) classes. For the HLA-associated phenotypes, we subdivided the HLA regions based on whether they fell into genes comprising the MHC Class I, MHC Class II regions or the remaining HLA region and then determined whether genetic liability for the 44 phenotypes was associated with these regions. For these analyses, a mixed model with four variance components was adopted: two comprised of SNPs located in and around genes corresponding to each MHC Class, respectively, one comprised of HLA region SNPs other than those in a MHC gene and one comprising the remaining SNPs. A phenotype was considered associated with a HLA class or region if the corresponding variance component had a *P*<0.05. A comparison of our class associations to those identified by prior GWAS studies (that is, a significant SNP association within the corresponding HLA regions was previously reported by GWAS) is shown in Table 1. All HLA class assignments, with one exception, either agreed with prior associations identified by GWAS or were not localized in our study. The exception was hypothyroidism, where we observed a Class II association not seen in prior GWAS, which had identified a Class I association.

We measured genetic correlations between the HLA-associated phenotypes shown in Table 1 to ascertain whether there was evidence of shared genetic risk among the phenotypes. Type 1 diabetes was significantly genetically correlated with both celiac disease (rG=0.84 (s.e. 0.50), *P*=0.00007, FDR *q*=0.007) and hypothyroidism (rG=0.31 (s.e. 0.12), *P*=0.003, FDR q=0.09), and juvenile rheumatoid arthritis was correlated with primary biliary cirrhosis (rG=0.55 (s.e. 0.21), *P*=0.003, FDR q=0.09) at a significance threshold of FDR *q*<0.1 (Supplementary Table 2).

### Exome chip association studies

To further explore these observations, we performed an exome chip association study for these six phenotypes with novel HLA localizations. No SNPs on the exome chip reached genome-wide significance using an additive logistic regression model for the sicca syndrome, dermatomyositis/polymyositis, dermatophytosis and cholangitis phenotypes (Supplementary Fig. 3).

**Figure 2:**
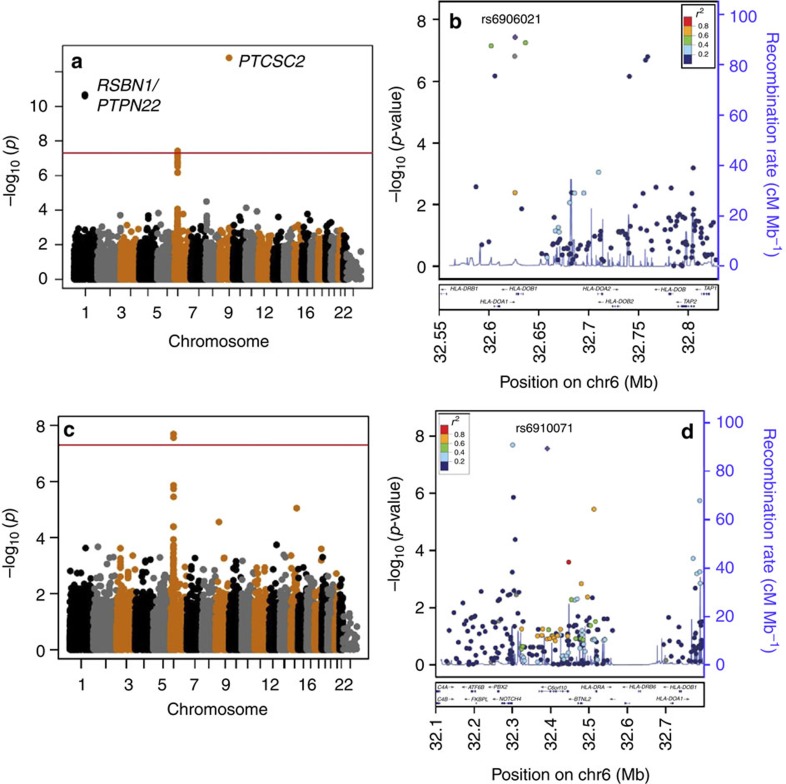
SNP association analysis for hypothyroidism and polymyalgia rheumatica. All analyses used an additive genetic model adjusted for three principal components, age and sex. (**a**) Manhattan plot for the hypothyroidism phenotype (3,242 cases, 6,484 controls) and (**b**) LocusZoom plot highlighting SNP rs6906021. (**c**) Manhattan plot for polymyalgia rheumatica (413 cases, 5,782 controls) and (**d**) LocusZoom plot highlighting SNP rs6910071.

Three SNPs reached genome-wide significance for hypothyroidism (Fig. 2a and Supplementary Figs 4 and 5). Two of the associations have been previously reported^3^,^24^: rs6679677 near *PHTF1/PTPN22* and rs965513 in the papillary thyroid cancer susceptibility candidate 2 (*PTCSC2*) gene. The third was a novel association at rs6906021 (chr6: odds ratio (OR)=1.2 (95% confidence interval (CI): 1.1–1.3), *P*=3.8 × 10^−8^) located near the HLA class II *HLA-DQA1* and *HLA-DQB1* genes (Fig. 2b and Table 2). Using data from the Marshfield Clinic Research Foundation, another EHR-based cohort, we were able to replicate this association (OR=1.2 (95% CI: 1.1–1.3), *P*=2.0 × 10^−4^), and meta-analysis gave a combined association of OR=1.2 (95% CI: 1.1–1.2), *P*=9.8 × 10^−11^ (Table 2).

For polymyalgia rheumatica (PMR), two SNPs in the HLA region reached genome-wide significance: rs3096702 (OR=1.5 (95% CI: 1.3–1.8), *P*=2.0 × 10^−8^) and rs6910071 (OR=1.6 (95% CI: 1.3–1.8), *P*=2.7 × 10^−8^) located upstream of *Notch4* and in *C6orf10*, respectively (Fig. 2c,d, Table 3 and Supplementary Figs 4 and 5). Only SNP rs6910071 replicated in the Marshfield data set: (OR=1.5 (95% CI: 1.2–2.0), *P*=0.002) (Table 3). The rs6910071 SNP was previously examined as part of a broad-based PheWAS study through the eMERGE network and was found to be associated with PMR (OR=1.2 (95% CI: 1.01–1.5), *P*=0.04)^19^. The meta-analyses for rs6910071 across data sets was significant (combined OR=1.5 (95% CI: 1.3–1.6), *P*=1.3 × 10^−10^) (Table 3).

In order to identify specific allelic variants associated with the SNP association signals, we tested for associations between the phenotypes and imputed classical 2- and 4-digit alleles at HLA-A, -B, -C, -DQA1, -DQB1 and -DRB1. For hypothyroidism, the strongest 4-digit allelic associations were seen at the HLA-DQA1*0501, HLA-DQB1*0201, HLA-DRB1*0301 alleles (*P*<10^−6^ for each allele), which collectively comprise the HLA-DR3-DQ2 haplotype (Supplementary Table 3). For polymyalgia rheumatic, the strongest allelic associations were with the HLA-DQA1*0301 and HLA-DRB1*04 alleles (*P*<10^−6^) (Supplementary Table 4).

## Discussion

We measured the genetic liability for 1,288 EHR-derived ICD-9-based phenotypes ascertained in a cohort of subjects with exome chip genotyping. We identified 44 HLA-associated phenotypes, five of which had not yet been studied by GWAS, and found that hypothyroidism was associated with SNP variation in the HLA class II region. This class II association was supported by the finding of a significant genetic correlation between hypothyroidism and Type 1 diabetes. Exome chip association analyses for hypothyroidism and PMR identified replicating SNP associations for both diseases in the HLA region.

One motivation for this analysis was to ascertain whether mixed models could identify phenotypes that had an underlying genetically mediated risk using rapidly assigned EHR phenotypes. By conducting the analyses using the highly selective set of SNPs captured on the Illumina exome chip platform, we were limited in the range of genetic variation we were able to interrogate. We found that the genetic liabilities were predominantly driven by SNPs located in the HLA region and with SNPs previously associated with phenotypes in the GWAS Catalog. This result would be expected as these SNPs have known biological significance and constitute a large proportion of the common SNP variation represented on the exome chip. Hence, we could not make broad interpretations about the global heritability of the phenotypes. However, the purpose of these analyses was to identify genetic phenotypes where further genetic characterization by a SNP association analysis using the SNPs on the exome chip platform could yield novel associations. In support of our analytical approach, we were able to reduce our set of 1,288 candidate phenotypes to six for which there was potential for novel SNP discovery on the exome chip. We were able to identify novel associations for two of these six phenotypes, hypothyroidism and PMR. Our general analytic approach would apply to any data set that genotyped common SNPs, even if the composition of SNPs on that platform were skewed toward distinct subsets. Application of this method to other large EHR-linked genotyped sets that are now becoming available, such as the Million Veterans Project, could yield robust heritability estimates across a much more diverse set of phenotypes.

The dense representation of common SNPs located within the HLA region provided a unique opportunity to identify phenotypes associated with this region. SNPs within this region were significant contributors to many of the most significant liability estimates. This is consistent with previous studies that have shown that a large portion of the genetic risk for autoimmune phenotypes is captured in this region^14^. We identified 44 phenotypes associated with this region at an FDR *q*<0.05. Eighteen of these phenotypes were specific autoimmune diseases, 13 of which were captured by the GWAS Catalog. Many of the other phenotypes represented clinical findings that could be consistent with an auto-immune aetiology but were not specific to any particular disease. Our inclusion criteria for this analysis may have been overly stringent, as there were a number of additional autoimmune diseases that fell slightly below this FDR threshold, including sarcoidosis, Crohn's disease, Grave's disease and uveitis, all of which have known HLA associations.

For most of the autoimmune diseases in the GWAS Catalog, we were able to assign their correct HLA class using mixed-modelling approaches. An exception was hypothyroidism, which had a class I association in the GWAS catalogue, and a weak class II association in our mixed models analysis. An exome chip association study identified a significant association near the class II *HLA-DQA1* and *HLA-DQB1* genes, suggesting that the class assignment by the mixed model was correct. Consistent with our findings, a prior GWAS found a suggestive SNP association in this same region, though the *P*-value did not reach genome-wide significance^24^. An HLA class II signal within this region is also supported by the significant genetic correlation we observed between hypothyroidism and type 1 diabetes, which has known HLA class II SNP associations, including *HLA-DQA1* (ref. 25). The pattern of HLA allelic associations for hypothyroidism was also suggestive of a HLA-DR3-DQ2 haplotype (DRB1*03:01-DQA1*05:01-DQB1*02), which is associated with type 1 diabetes and coeliac disease^26^. Clinically, hypothyroidism and type 1 diabetes co-occur frequently^27^.

PMR was also associated with SNP variation in the HLA region. PMR is a steroid-responsive autoimmune disease of the musculature that typically presents in adults over 50 years old^28^. We identified two SNPs associated with PMR that reached genome-wide significance. One SNP, rs6910071, located in *C6orf10* has previously been associated with the autoimmune disease rheumatoid arthritis^29^. The second SNP has no reported associations, and did not replicate in our analyses. HLA typing studies have shown that PMR is associated with HLA gene allelic variants in the *HLA-DR* region and the HLA-DR4 antigen^30^,^31^. Hence, the rs6910071 SNP may be tagging this haplotype.

This study has limitations. Of primary importance, the exome chip contains a highly selected panel of SNPs and most common SNPs across the genome are not covered on the platform. The limited representation of common SNPs on the exome chip, beyond those in the GWAS catalogue and the HLA region, makes it difficult to ascertain whether a low-significance genetic risk estimate is the result of a poor-specificity phenotype definition or a lack of relevant SNPs included on the exome chip platform. However, within the context of the primary aims of this analysis, a non-significant genetic liability estimate indicated that pursuing the phenotype further based on the SNPs available on the exome chip was unlikely to identify novel associations. To control for stratification and cryptic relatedness, we either included a large number of principal components (PCs) or, when possible, used a second genetic relationship matrix (GRM)^17^ in our data models. Finally, for many of the phenotypes, there were a relatively small number of cases and, hence, we may have been underpowered to measure a genetic component for these phenotypes. This may have contributed to our inability to identify significant associations for four of the auto-immune diseases that we evaluated by a SNP association study. In addition, we observed that low case counts were more frequently associated with negative liability estimates, suggesting that there may be more false-positive findings among phenotypes with few cases.

In summary, we used mixed models to analyse the genetic risk underlying a large set of EHR-derived phenotypes and were able to efficiently identify a subset of genetically modulated phenotypes that led to the identification of novel SNP-phenotype associations. Hence, these analyses demonstrate the utility of the mixed-models approach to identify and broadly classify genetic phenotypes.

## Methods

### Study population

The study population comprised 29,349 previously genotyped adult and paediatric subjects of genetic EA identified through BioVU, a de-identified collection of patients whose DNA was extracted from discarded blood and linked to phenotypes through a de-identified electronic medical record^32^. The study subjects were genotyped on the Illumina Human Exome Beadchip v1.1 as part of a broad-based genotyping initiative. Genetic ancestry assignment was determined using STRUCTURE^33^ in conjunction with 2,652 ancestry informative markers, with EA defined as >90% probability of being in the HapMap CEU cluster.

### Ethics statement

The Vanderbilt BioVU resource operates as nonhuman subjects research according to the provisions of the 45 *Code of Federal Regulations*, part 46, with oversight by Vanderbilt's Institutional Review Board, as previously described^32^. This project was deemed nonhuman subjects by the Vanderbilt Institutional Review Board.

### Phenotype data

The phenotypes evaluated in this study were based on PheWAS codes (phenotypes), which are collections of related ICD-9 codes^6^,^19^,^20^,^34^. ICD-9 codes capture clinical data related to the diagnoses, signs and symptoms, and history of disease. PheWAS phenotypes have a hierarchical structure, such that some phenotypes are comprised of collections of other phenotypes that represent diseases with similar clinical characteristics. For instance, the ‘Diabetes Mellitus' code incorporates both Type 1 and Type 2 diabetes and has ‘children' concepts of Types 1 and 2 diabetes. For each PheWAS phenotype, cases are subjects with two or more instances of the PheWAS code appearing in their medical record on separate dates^19^. Birth decade- and sex-frequency-matched controls with no instances of that code are randomly selected for each PheWAS phenotype There are over 1,600 defined PheWAS codes, of which 1,288 had 50 or more cases and were used in these analyses. PheWAS phenotypes and their respective ICD-9 mappings are available at http://PheWAScatalog.org. PheWAS phenotypes were manually mapped to the NHGRI GWAS Catalog phenotypes (http://www.genome.gov/gwastudies/), as previously described^19^. A subset of phenotypes with a significant genetic liability component (described below) were reviewed by a clinician and classified as to whether they had a known autoimmune aetiology (shown in Supplementary Data 1).

### Genotyping data

Genotype data were acquired on the Illumina Infinium Exome BeadChip v1.1. A subset of individuals was also genotyped on the OMNI HumanOmni1-Quad and HumanOmni5-Quad platforms^35^. The OMNI genotyping data were used only to establish relatedness thresholds, as described below. Quality control was performed on the Exome BeadChip data by VANGARD (Vanderbilt Technologies for Advanced Genomics Analysis and Research Design) using Genome Studio and PLINK, as previously described^36^,^37^. Briefly, SNPs were clustered using Genome Studio, and to ensure correctness, manual reclustering was performed based on quality control measurements such as GenTrain Scores, Cluster Separation, Call Freq scores. Samples were evaluated for heterozygous consistency rates between duplicated samples, heterozygous consistency rate between HAPMAP samples and their 1000 Genome genotyping calls, sex mismatches, and genotype consistency between duplicated SNPs on the SNP chip. After QC, there were 33,233 SNPs with a MAF>1% and a Hardy-Weinberg *P*>0.001. PCs were computed using EIGENSTRAT^38^ to adjust for residual population structure. Intensity plots and quality control statistics for the three SNPs in the HLA region with novel findings in these analyses are shown in Supplementary Fig. 6.

### Primary analyses

Generalized linear mixed models (GLMMs) were used to estimate the global genetic contributions of SNP variation to the underlying variation of each phenotype. We employed the GLMM algorithms implemented in the Genome-wide Complex Trait Analysis program^11^,^15^,^39^,^40^,^41^. First, a GRM was computed using autosomal SNPs with a MAF>1%. Due to the small number of highly selected SNPs represented on the exome chip, when the recommended relatedness exclusion threshold of 0.025 was applied^15^,^42^, almost all subjects were excluded. In order to empirically determine a relatedness cut-off appropriate to the Exome chip, we examined a subset of 4,564 subjects who also had genome-wide genotyping on OMNI HumanOmni1-Quad and HumanOmni5-Quad platforms and who had a genome-wide relatedness score less than 0.025 on these genome-wide SNP platforms. We then computed a GRM for these subjects using the exome chip in conjunction with a LD-reduced set of 6,541 SNPs. The upper limit of the relatedness score in these subjects was 0.07. When we applied this threshold to the entire exome data set, 3,154 subjects were removed (10.7%), a proportion consistent with the exclusion rates that we have observed in analyses using genome-wide SNP genotyping involving data derived from other analyses using the Vanderbilt University EHR.

Genetic liability estimates for each PheWAS phenotype were computed using a GLMM in conjunction with a GRM derived from exome chip SNPs. Estimates were adjusted for age, sex and 20 PCs. *P*-values were computed by a likelihood ratio test (LRT) comparing a full model with a model excluding the GRM. FDR-adjusted *P*-values (*q*-values) were determined using a Benjamini-Hochberg adjustment. Genetic liability estimates for each phenotype were also computed using a GRM that excluded 2,147 SNPs located in the chromosomal region surrounding the HLA region on chromosome 6 (chr6:26,000,000–34,000,000), and using a GRM that further excluded 4,641 SNPs representing 2,199 SNPs from the GWAS catalogue with a phenotype association at *P*<5 × 10^−8^ and exome chip SNPs with an *r*^2^>0.2 with any of these SNPs.

In order to identify phenotypes associated with the HLA region, a mixed model employing two GRMs, one based on SNPs in the HLA region and the other based on all remaining SNPs, and partitioning the genetic variance between the GRMs was used. The *P*-value associated with the HLA GRM was computed using a LRT comparing the full model (both GRMs) to a model that excluded the HLA GRM. Those diseases with a FDR *q*<0.05 were further evaluated. These phenotypes were further evaluated to identify the HLA class-association regions associated with the phenotype. For each phenotype, a model that incorporated four GRMs (three related to HLA SNP subgroups and a fourth for the remaining non-HLA SNPs) was fit. The three HLA subgroups comprised all Exome chip SNPs located within 25 kb of MHC Class I genes (*HLA-A*, *-B*, *-C*, *-E*, *-F* and *-G* genes) (*n*=141 SNPs); MHC Class 2 genes (*HLA-DP*, *-DQ*, *-DR*, *-DM* and *-DO* genes) (*n*=208 SNPs), and all other genes located in the HLA region (*n*=1,698 SNPs). A *P*-value for each HLA subgroup was determined by dividing the square of the (genetic liability estimate divided by its standard error) and calculating the corresponding *P*-value assuming a chi-square distribution with 1 degree of freedom. Where possible, the results of these analyses were compared to associations reported in the NHGRI GWAS catalogue by manually mapping each PheWAS code to a disease in the GWAS catalogue and identifying the genes within the respective MHC regions that contained SNPs with association *P*-values <5 × 10^−8^.

Genetic correlations were computed for pairs of phenotypes associated with the HLA region and that had mapped to a GWAS catalogue phenotype by fitting a bivariate GLMM, adjusting for age, gender and 20 PCs. *P*-values were determined by computing the log likelihood from full model (L1) and a model where the genetic correlation was constrained to be 0 (L0). The *P*-value was based on a LRT −2(L1−L0), assuming a chi-square distribution with 1 degree of freedom.

To identify SNPs associated with the hypothyroidism and PMR phenotypes, an association study employing an additive logistic model and adjusting for age, sex and three PCs was run using all SNPs on the exome chip with MAF>1%. A *P*-value below *P*<5 × 10^−8^ was considered genome-wide significant.

### Replication analyses

Significant SNP associations within the HLA region were replicated using data from 10,124 PMRP patients in the Personalized Medicine Research Project (PMRP)^43^,^44^, which has been used for PheWAS analysis previously^7^,^9^,^45^. Use of the Marshfield data was approved by the Marshfield Clinic Research Foundation Institutional Review Board. All individuals genotyped are self-reported white/non-Hispanic, with over 70% claiming German ancestry. The PMRP subjects are 63% female, with a mean age of 65 years and having on average over 30 years of clinic data in Marshfield Clinic's EHR system. Subjects were genotyped on the Illumina HumanCoreExome BeadChip as part of a case-control study of AMD. PMR and hypothyroidism phenotypes in this data set were derived using the same ICD-9 codes as the primary analyses, defined by the PheWAS code mappings. Replications were tested using an additive logistic model and adjusting for age and sex. An association *P*<0.05 was considered significant. In addition, one significantly associated SNP (rs6910071) had been previously evaluated by a PheWAS as part of the eMERGE consortium and its association with PMR was obtained from the publicly available PheWAS catalogue (http://phewas.mc.vanderbilt.edu/)^19^.

### HLA allele imputation

HLA alleles were imputed from SNP data on the HumanExome BeadChip using SNP2HLA with the Type 1 Diabetes Genetics Consortium reference panel and a marker window size of 1,000 (ref. 46). We imputed in each of the samples individual dosages for 298 classical 2- and 4-digit alleles at HLA-A, -B, -C, -DQA1, -DQB1 and -DRB1. A total of 173 classical 4-digit HLA alleles included in the analysis had a MAF>1%. Association testing for each allele and the polymyalgia and hypothyroidism phenotypes was performed using an additive logistic model and adjusting for age, sex and three PCs.

### Data analysis

All quality control analyses and SNP association analyses were performed using PLINK v1.07 (ref. 47). Genetic liability estimates were computed using the Genome-wide Complex Trait Analysis v1.24 (ref. 39). All other analyses were performed using SAS v9.3 (SAS Institute, Cary, NC). SNP data were visualized using LocusZoom^48^. HLA allele imputation was performed using SNP2HLA^46^.

## Additional information

**How to cite this article:** Mosley, J. D. *et al*. Identifying genetically driven clinical phenotypes using linear mixed models. *Nat. Commun.* 7:11433 doi: 10.1038/ncomms11433 (2016).

## Supplementary Material

Supplementary Information Supplementary Figures 1-6 and Supplementary Tables 1-4

Supplementary Data 1 Genetic liability estimates for PheWAS phenotypes using a mixed model and a GRM containing all autosomal SNPs on the Exome chip.

Supplementary Data 2 Genetic liability estimates for SNPs located within the HLA genomic region.

## Figures and Tables

**Table 1:** PheWAS phenotypes associated with SNP variation in the HLA region*.

**PheWAS disease**	**Genetic liability (s.e.)**†	***P*****-value**†	**Class I**‡	**Class II**‡	**Other HLA**‡
*Known associations*					
Ankylosing spondylitis	0.074 (0.013)	<1.0 × 10^−20^	0.0006§	1	0.012
Type I diabetes	0.099 (0.014)	<1.0 × 10^−20^	1	0.00002§	0.0033§
Rheumatoid arthritis	0.035 (0.008)	<1.0 × 10^−20^	1§	0.005§	0.041§
Multiple sclerosis	0.023 (0.006)	<1.0 × 10^−20^	0.68§	0.01§	0.23§
Hypothyroidism	0.009 (0.003)	5.6 × 10^−9^	0.7§	0.067	0.13§
Psoriasis	0.017 (0.005)	6.5 × 10^−9^	0.044§	0.97	0.17
Juvenile rheumatoid arthritis	0.025 (0.008)	1.1 × 10^−8^	0.21	0.026§	0.42
Primary biliary cirrhosis	0.017 (0.006)	6.7 × 10^−8^	0.41	0.057§	0.19
Coeliac disease	0.008 (0.004)	3.8 × 10^−7^	1	0.062§	0.31
Macular degeneration	0.013 (0.005)	3.2 × 10^−5^	1	1	0.014§
Ulcerative colitis||	0.007 (0.003)	3.9 × 10^−5^	n/a	n/a§	n/a§
Systemic lupus erythematosus	0.005 (0.003)	2.7 × 10^−4^	0.89	0.59§	0.22§
Premature menopause	0.006 (0.003)	1.0 × 10^−3^	0.72	0.72	0.25§
					
*Novel associations*					
Sicca syndrome	0.009 (0.004)	1.8 × 10^−6^	1	0.14	0.28
Dermatomyositis and polymyositis	0.008 (0.004)	1.1 × 10^−5^	0.11	0.88	0.61
Polymyalgia rheumatica	0.010 (0.005)	3.5 × 10^−4^	1	0.8	0.042
Cholangitis	0.006 (0.004)	5.8 × 10^−4^	0.38	1	0.17
Dermatophytosis of the body	0.004 (0.003)	6.8 × 10^−4^	1	1	0.16

^*^Only phenotypes with an FDR *q*<0.05 and that mapped to the GWAS Catalog or have unreported associations are shown.

^†^From a multivariable GLMM analysis that incorporated a HLA and non-HLA GRM and adjusted for age and sex each of the PheWAS phenotypes. *P*-values correspond to the HLA variance component.

^‡^Shown are *P*-values from a multivariable GLMM analysis incorporating GRMs comprising SNPs within the specificied HLA region (see Methods).

^||^An estimate could not be obtained because the statistical model did not converge.

^§^A SNP association with a *P*-value<5 × 10^−8^ in the GWAS Catalog reported in the specified region.

**Table 2:** Significant SNP associations and replication of HLA-associated SNPs for hypothyroidism.

**Data set/SNP**	Location*	**Reference allele**	**MAF cases/controls**	**OR**	**95% CI**	***P*****-value**
*Discovery cohort† (3,242 cases, 6,484 controls)*						
rs6679677	chr1:113761186	A	0.12/0.09	1.39	1.25–1.53	2.5 × 10^−11^
rs2476601	chr1:113834946	A	0.12/0.09	1.39	1.26–1.53	2.1 × 10^−11^
rs6906021	chr6:32658534	C	0.49/0.44	1.19	1.12–1.27	3.8 × 10^−8^
rs965513	chr9:97793827	A	0.30/0.35	0.78	0.73–0.84	1.6 × 10^−13^
						
*Marshfield replication‡ (1,645 cases, 7,127 controls)*						
rs6906021	chr6:32658534	C	0.49/0.46	1.16	1.07–1.26	2.0 × 10^−4^
						
*Meta-analysis*						
rs6906021	chr6:32658534	C		1.18	1.12–1.24	9.8 × 10^−11^

^*^Coordinates are for genome assembly GRCh38.p2.

^†^From an additive logistic regression model, adjusting for age, gender and three principal components.

^‡^From an additive logistic regression model, adjusting for age and gender.

**Table 3:** Significant SNP associations and replication of HLA-associated SNPs for polymyalgia rheumatica.

**Data set/SNP**	Location*	**Reference allele**	**MAF cases/controls**	**OR**	**95% CI**	***P*****-value**
*Discovery cohort† (413 cases, 5,782 controls)*						
rs3096702	chr6:32224554	T	0.48/0.39	1.52	1.31–1.76	2.0 × 10^−8^
rs6910071	chr6:32315077	G	0.30/0.22	1.58	1.34–1.85	2.7 × 10^−8^
						
*PheWAS Catalog‡ (273 cases, 13,464 controls)*						
rs6910071	chr6:32315077	G	n/a	1.24	1.01–1.52	0.04
						
*Marshfield replication§ (146 cases, 9,838 controls)*						
rs3096702	chr6:32224554	T	0.40/0.36	1.20	0.84–1.52	0.14
rs6910071	chr6:32315077	G	0.26/0.18	1.53	1.17–2.0	0.002
						
*Meta-analysis*						
rs6910071				1.46	1.30–1.63	1.3 × 10^−10^

^*^Coordinates are for genome assembly GRCh38.p2.

^†^From an additive logistic regression model, adjusting for age, gender and three principal components (PCs).

^‡^Results extracted from the PheWAS Catalog for an additive logistic regression model, adjusting for age, gender and three PCs.

^§^From an additive logistic regression model, adjusting for age and gender.
