# Supplementary material for: Identifying genetically driven clinical phenotypes using linear mixed models
Source: Nat Commun. 2016 Apr 25;7:11433. doi: 10.1038/ncomms11433 (PMC4848547; doi:10.1038/ncomms11433)
Supplement: Supplementary Information — Supplementary Figures 1-6 and Supplementary Tables 1-4 [file ncomms11433-s1.pdf]

Supplementary figure 1

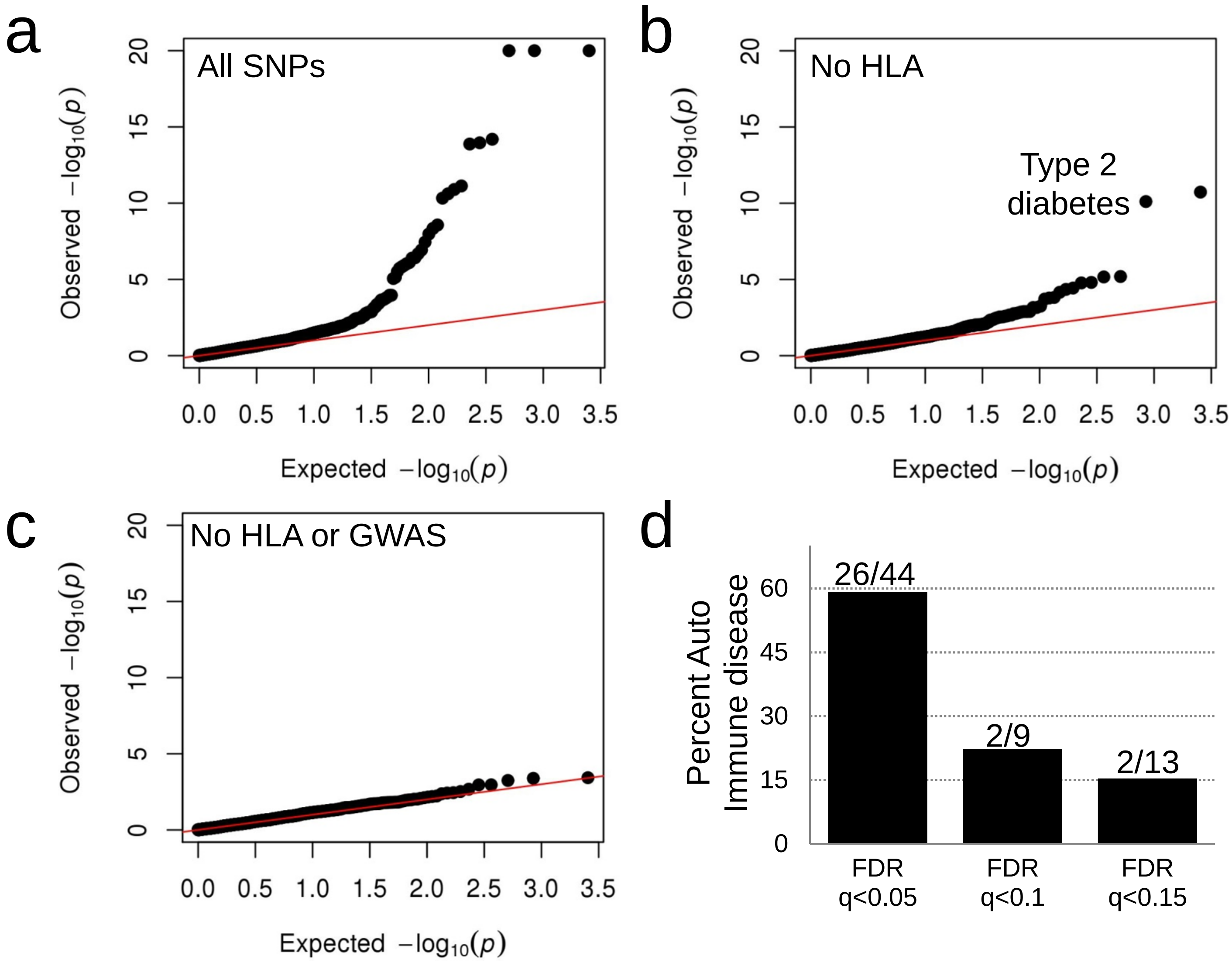

Supplementary Figure 1. HLA-associated SNPs underlie significant liability estimates. a-c) QQ plots where each point represents a mixed models analysis of a phenotype, adjusted for age, sex and 20 principal components a) using all SNPs with MAF>1%, b) excluding HLA region SNPs or c) exclude HLA region and SNPs with significant associations in the GWAS catalog. PheWAS phenotypes with p-values<10^{-20} (3 phenotypes) were set to 10^{-20} for display purposes. d) Each bar represents the proportion of phenotypes that are known autoimmune diseases binned by FDR q-value thresholds for a multivariable GLMM analysis that incorporated a HLA and non-HLA GRM and adjusted for age and sex each PheWAS phenotypes. P-values were determined using a likelihood ratio test comparing the 2 GRM model to a model that only included the non-HLA GRM. The numbers above the bars are the number of autoimmune disease and total phenotypes for each bin.

. .

Supplementary figure 2

PheWAS disease code	Class I	Class II	Other HLA
Dermatophytosis of the body			
Hypothyroidism			
**Hypothyroidism NOS**	*HLA-B, HLA-C*		*C6orf15, HCG22, HCP5*
Diabetes mellitus			
**Type 1 diabetes**		*HLA-DRB1*	*PHF1*
Type 1 diabetes with ketoacidosis			
Type 1 diabetes with renal manifestation			
Type 1 diabetes with ophthalmic manifest			
Type 1 diabetes with neurological manifest			
Insulin pump user			
Diabetic retinopathy			
Hypoglycemia			
**Multiple sclerosis**	*HLA-B*	*HLA-DQB1,HLA-DRA,HLA-DRB1*	*C6orf10*
Other demyelinating diseases of central			
Lack of coordination			
**Macular degeneration (senile) of retina**			*C2, CFB, DDR1, IER3, NOTCH4, SKIV2L, STK19, TNXB*
Esophageal bleeding (varices/hemorrhage)			
Other disorders of stomach and duodenum			
**Inflammatory bowel diseases**		*HLA-DQA1*	
**Ulcerative colitis**		*HLA-DQA1,HLA-DQB1,HLA-DRA,HLA-DRB1,HLA-DRB5*	*BTNL2*
**Celiac disease**		*HLA-DQA1, HLA-DQB1*	
Chronic liver disease and cirrhosis			
Other chronic nonalcoholic liver disease			
Cirrhosis of liver without mention of alcohol			
**Primary biliary cirrhosis**		*HLA-DQB1*	
Liver abscess and sequelae of liver disease			
Portal hypertension			
Abnormal results of function study of liver			
Cholangitis			
**Premature menopause and ovarian failure**			*PRR2CA*
Lupus			
**Systemic lupus erythematosus**		*HLA-DQA1,HLA-DQA2,HLA-DRB1*	*TNXB*
Psoriasis and related disorders			
**Psoriasis**	*HLA-C*		
**Psoriasis vulgaris**	*HLA-C*		
**Psoriatic arthropathy**	*HLA-C*		
**Diffuse diseases of connective tissue**			
Sicca syndrome			
Dermatomyositis and Polymyositis			
Rheumatoid arthritis and other diseases			
**Rheumatoid arthritis**	*HLA-G*	*HLA-DQA1,HLA-DQA2,HLA-DQB1,HLA-DRA,HLA-DRB1*	*APOM,C6orf10,MICA*
**Juvenile rheumatoid arthritis**		*HLA-DRB1*	
**Ankylosing spondylitis**	*HLA-B*		
Polymyalgia Rheumatica			

Supplementary figure 2

Supplementary Figure 2. PheWAS phenotypes associated with SNP variation in the HLA region. Genetic liabilities were computed using a multivariable mixed model that incorporated a HLA and non-HLA GRM and adjusted for age and sex each PheWAS codes. P-values were determined using a likelihood ratio test comparing the 2 GRM model to a model that only included the non-HLA GRM. Phenotypes with an FDR p-value<0.05 were selected. PheWAS codes are shown by hierarchy and progressive indentation indicates that the child (indented) phenotype contains a subset of subjects from the parent (unindented) phenotype. Color highlighting indicates that a phenotype was associated with SNP variation located within selected HLA regions at either a p<0.05 (green) or p<0.1 (yellow) of significance using a multi GRM mixed model (see Methods). Red highlighting indicates that an estimate could not be obtained for the PheWAS code.

Supplementary figure 3

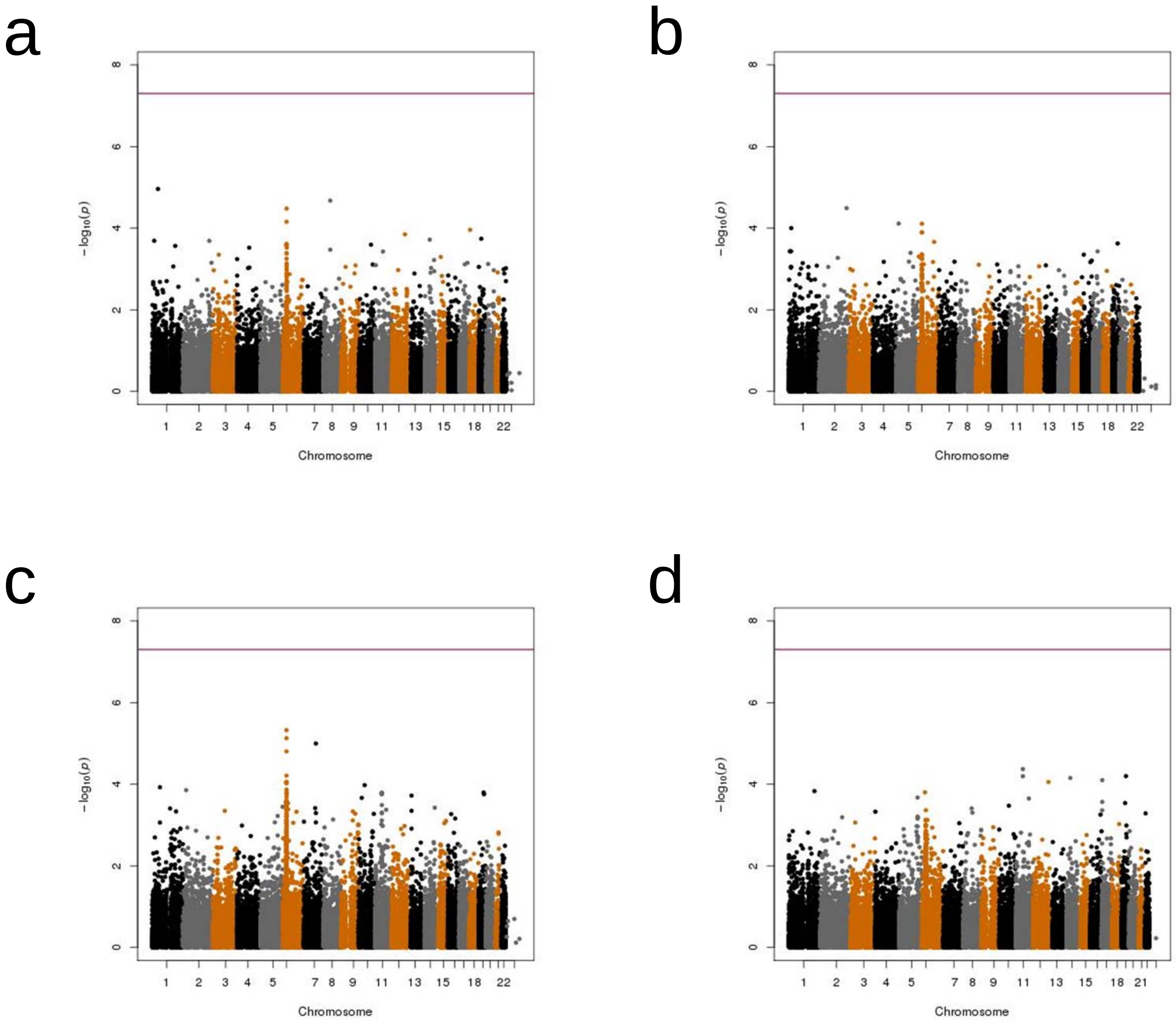

Supplementary Figure 3. Manhattan plots using an additive genetic model for a) Cholangitis (245 cases, 5,880 controls), b) Dermatophytosis (90 cases, 5,850 controls), c) Polymyositis/dermatomyositis (178 cases, 5,882 controls) and d) Sicca syndrome (157 cases, 5,809 controls), adjusted for 3 principal components, age and sex. .

Supplementary figure 4

a b

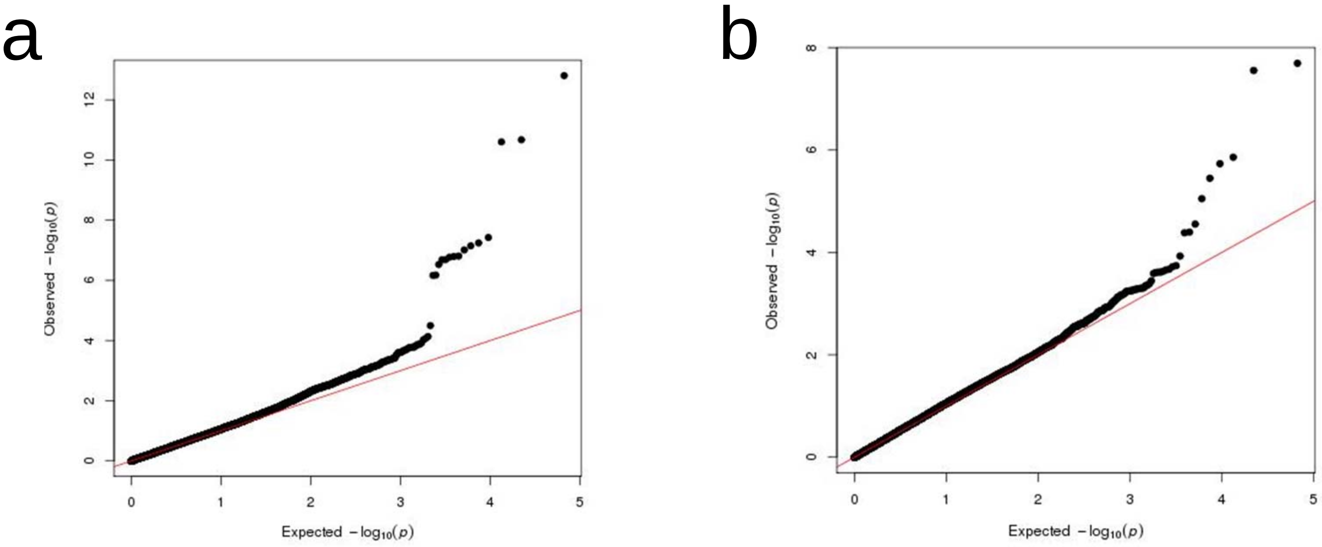

Supplementary Figure 4. QQ plots using an additive genetic model for the a) hypothyroidism phenotype (3242 cases, 6484 controls) and b) Polymyalgia rheumatica (413 cases, 5782 controls), adjusted for 3 principal components, age and sex.

Supplementary figure 5

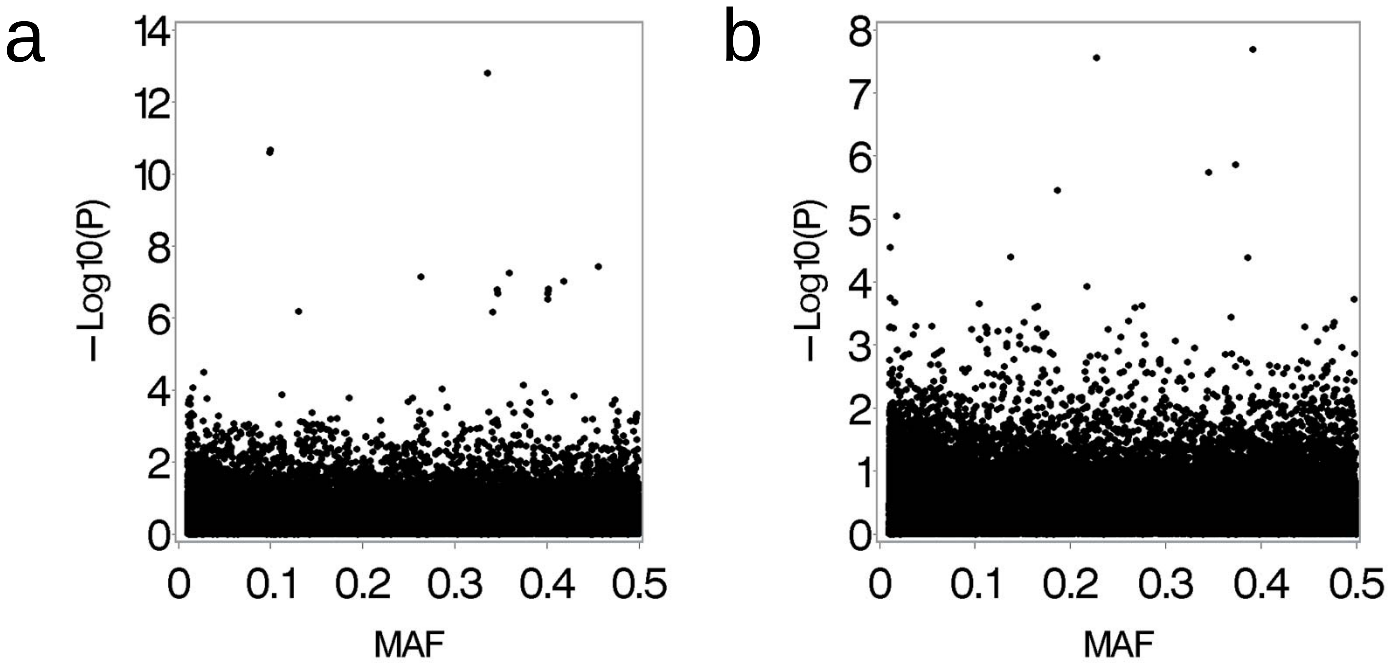

Supplementary Figure 5. Plot of p-values versus minor allele frequencies for a) hypothryoidism phenotype (3242 cases, 6484 controls) and b) Polymyalgia rheumatica (413 cases, 5782 controls), adjusted for 3 principal components, age and sex.

Supplemental figure 6

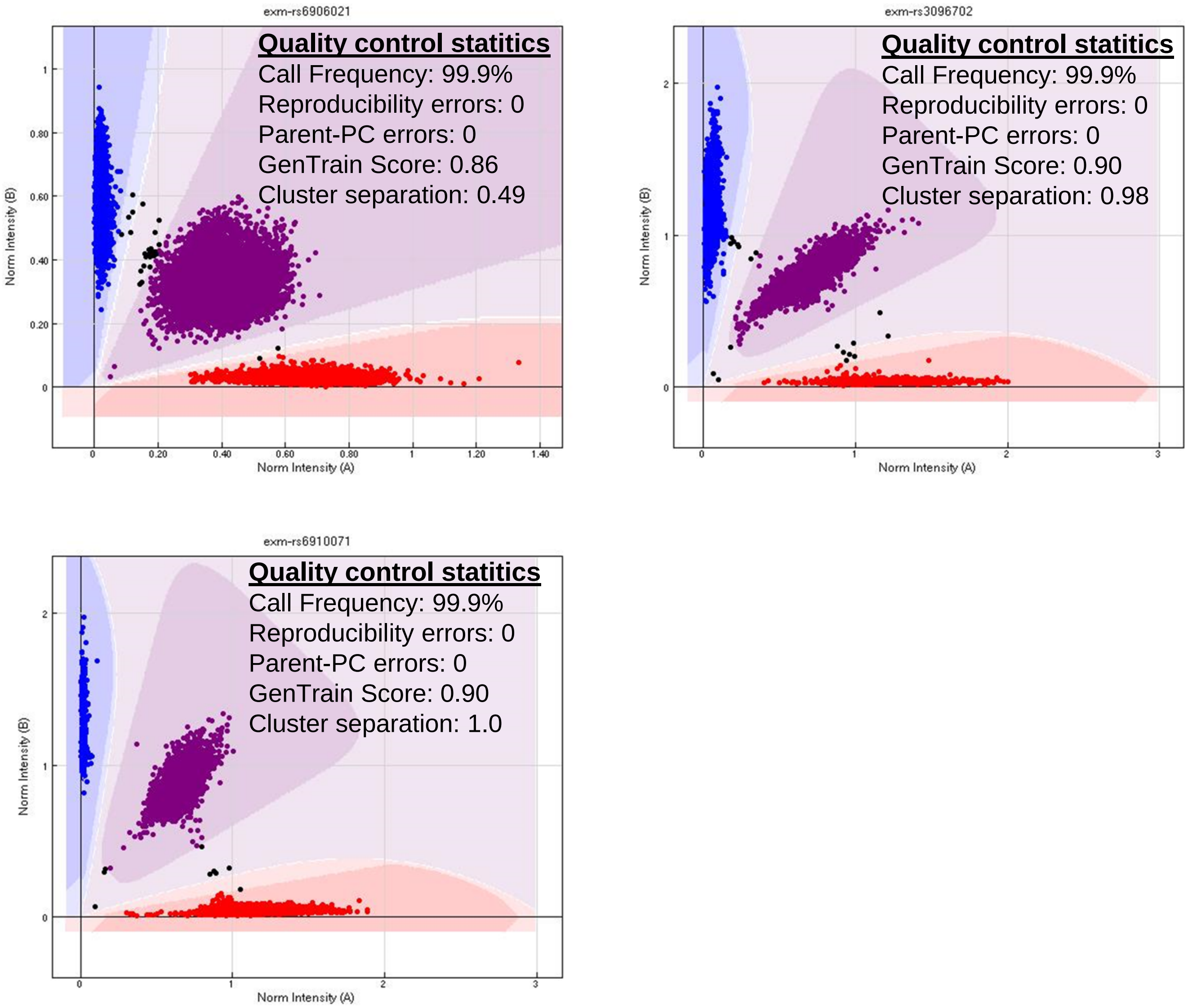

Supplementary Figure 6. Intensity plots for 3 SNPs in the HLA region associated with polymyalgia rheumatica or hypothyroidism. Points in black indicate that a call was not made. Shown also are select quality control statistics for each SNPs. During genotyping of the cohort, there were ~1,039 HAPMAP sample platings (representing 36 distinct individuals) and there was a 99.7% concordance to the HAPMAP reference genotype for these 3 HLA SNPs.

Supplementary Table 1. Characteristics of the study population.

Characteristic	n	%
Males	13,668	46.6
Females	15,681	53.4
Age < 18	3,842	13.1
Age ≥ 18	25,507	86.9
**Average years of follow-up**		
median (IQR)	8	(4-14)
**Distinct PheWAS codes per subject**		
median (IQR)	22	(10 - 41)
**Most frequent diagnoses**		
Hypertension	11,675	
Essential hypertension	11,478	
Disorders of lipid metabolism	8,411	
Hyperlipidemia	8,381	
Malaise and fatigue	7,644	
Pain in joint	7,405	
Cardiac dysrhythmias	7,338	
Abdominal pain	7,199	
Disorders of fluid, electrolyte, and acid balance	6,992	
Other anemias	6,519	
Diseases of esophagus	5,732	
Ischemic Heart Disease	5,317	
Nausea and vomiting	5,187	
Esophagitis, GERD and related diseases	5,134	
Electrolyte imbalance	4,948	
Diabetes mellitus	4,842	
Anemia	4,798	
Fever of unknown origin	4,763	
Coronary atherosclerosis	4,739	
GERD	4,574	
Osteoporosis, osteopenia and pathological fracture	4,437	
Renal failure	4,275	
Type 2 diabetes	4,227	
Urinary tract infection	4,195	
Mixed hyperlipidemia	4,112	

Osteoarthrosis	4,110
Other symptoms/disorders or the urinary	4,059
Mood disorders	4,058

Supplementary Table 2. Genetic correlations between pairs of phenotypes associated with SNP variation in the HLA region. Estimates were obtained using a bivariate model, adjusted for age, sex and 20 principal components. P-values were based on a likelihood ratio test compared to a model where the genetic correlation was fixed at 0.

Phewas Code	Code description	Phewas Code	Code description	Genetic correlation	Standard error	Likelihood ratio test statistic (-2LogL)	p-value	FDR q-value
557.1	Celiac disease	250.1	Type 1 diabetes	0.84	0.50	15.7	0.00007	**0.007**
250.1	Type 1 diabetes	244.4	Hypothyroidism NOS	0.31	0.12	9.0	0.003	**0.093**
714.2	Juvenile rheumatoid arthritis	571.6	Primary biliary cirrhosis	0.55	0.21	8.8	0.003	**0.093**
714.1	Rheumatoid arthritis	571.6	Primary biliary cirrhosis	0.37	0.16	6.6	0.01	0.226
695.4	Lupus (localized and systemic)	362.29	Macular degeneration (senile) of retina NOS	-0.59	0.27	5.6	0.02	0.323
709.4	Polymyositis	335	Multiple sclerosis	-0.45	0.24	4.6	0.03	0.475
571.6	Primary biliary cirrhosis	250.1	Type 1 diabetes	0.21	0.12	4.1	0.04	0.496
555.2	Ulcerative colitis	250.1	Type 1 diabetes	-0.28	0.17	4.1	0.04	0.496
709.4	Polymyositis	709.2	Sicca syndrome	1.00	0.86	3.8	0.05	0.508
695.4	Lupus (localized and systemic)	555.2	Ulcerative colitis	0.62	0.37	3.7	0.06	0.508
709.2	Sicca syndrome	557.1	Celiac disease	1.00	0.76	3.4	0.06	0.537
714.1	Rheumatoid arthritis	250.1	Type 1 diabetes	0.16	0.11	2.9	0.09	0.622
555.2	Ulcerative colitis	362.29	Macular degeneration (senile) of retina NOS	-0.40	0.26	2.8	0.10	0.622
709.2	Sicca syndrome	695.4	Lupus (localized and systemic)	0.56	0.33	2.8	0.10	0.622
695.4	Lupus (localized and systemic)	335	Multiple sclerosis	0.36	0.26	2.7	0.10	0.622
714.2	Juvenile rheumatoid arthritis	335	Multiple sclerosis	-0.26	0.18	2.6	0.11	0.622
715.2	Ankylosing spondylitis	714.2	Juvenile rheumatoid arthritis	0.24	0.16	2.4	0.12	0.622
709.4	Polymyositis	695.4	Lupus (localized and systemic)	0.57	0.41	2.3	0.13	0.622
717	Polymyalgia Rheumatica	714.1	Rheumatoid arthritis	0.26	0.19	2.3	0.13	0.622
557.1	Celiac disease	244.4	Hypothyroidism NOS	0.41	0.32	2.1	0.15	0.678
715.2	Ankylosing spondylitis	695.4	Lupus (localized and systemic)	-0.29	0.23	2.0	0.16	0.701
709.4	Polymyositis	557.1	Celiac disease	0.66	0.63	1.7	0.19	0.708
709.2	Sicca syndrome	335	Multiple sclerosis	0.25	0.21	1.7	0.19	0.708
571.6	Primary biliary cirrhosis	244.4	Hypothyroidism NOS	0.23	0.18	1.7	0.19	0.708
714.2	Juvenile rheumatoid arthritis	709.2	Sicca syndrome	0.33	0.28	1.7	0.19	0.708
717	Polymyalgia Rheumatica	709.4	Polymyositis	0.31	0.29	1.3	0.25	0.822
709.2	Sicca syndrome	244.4	Hypothyroidism NOS	0.28	0.26	1.3	0.26	0.822
709.4	Polymyositis	571.6	Primary biliary cirrhosis	0.30	0.29	1.3	0.26	0.822
362.29	Macular degeneration (senile) of retina NOS	250.1	Type 1 diabetes	-0.12	0.11	1.3	0.26	0.822
715.2	Ankylosing spondylitis	362.29	Macular degeneration (senile) of retina NOS	-0.16	0.15	1.2	0.28	0.828
709.4	Polymyositis	250.1	Type 1 diabetes	0.16	0.17	1.2	0.28	0.828
557.1	Celiac disease	362.29	Macular degeneration (senile) of retina NOS	-0.31	0.33	1.1	0.30	0.841
709.4	Polymyositis	362.29	Macular degeneration (senile) of retina NOS	0.24	0.26	1.0	0.32	0.886
555.2	Ulcerative colitis	244.4	Hypothyroidism NOS	0.19	0.22	0.8	0.36	0.912

362.29	Macular degeneration (senile) of retina NOS	335	Multiple sclerosis	-0.14	0.16	0.8	0.36	0.912
709.2	Sicca syndrome	362.29	Macular degeneration (senile) of retina NOS	-0.22	0.26	0.8	0.38	0.912
709.4	Polymyositis	244.4	Hypothyroidism NOS	0.20	0.26	0.7	0.41	0.912
714.2	Juvenile rheumatoid arthritis	714.1	Rheumatoid arthritis	0.13	0.17	0.7	0.41	0.912
555.2	Ulcerative colitis	335	Multiple sclerosis	0.16	0.21	0.7	0.41	0.912
715.2	Ankylosing spondylitis	244.4	Hypothyroidism NOS	-0.10	0.13	0.6	0.43	0.912
362.29	Macular degeneration (senile) of retina NOS	244.4	Hypothyroidism NOS	0.13	0.18	0.6	0.45	0.912
717	Polymyalgia Rheumatica	557.1	Celiac disease	-0.26	0.37	0.6	0.46	0.912
709.2	Sicca syndrome	250.1	Type 1 diabetes	0.10	0.16	0.5	0.46	0.912
715.2	Ankylosing spondylitis	709.4	Polymyositis	-0.15	0.22	0.5	0.46	0.912
717	Polymyalgia Rheumatica	362.29	Macular degeneration (senile) of retina NOS	0.15	0.21	0.5	0.46	0.912
714.1	Rheumatoid arthritis	695.4	Lupus (localized and systemic)	0.14	0.21	0.5	0.48	0.912
714.1	Rheumatoid arthritis	709.2	Sicca syndrome	0.13	0.21	0.4	0.53	0.912
714.2	Juvenile rheumatoid arthritis	250.1	Type 1 diabetes	-0.07	0.12	0.4	0.53	0.912
717	Polymyalgia Rheumatica	250.1	Type 1 diabetes	0.07	0.12	0.3	0.56	0.912
714.2	Juvenile rheumatoid arthritis	244.4	Hypothyroidism NOS	-0.10	0.18	0.3	0.57	0.912
715.2	Ankylosing spondylitis	709.2	Sicca syndrome	-0.11	0.21	0.3	0.57	0.912
714.1	Rheumatoid arthritis	244.4	Hypothyroidism NOS	0.08	0.15	0.3	0.57	0.912
714.2	Juvenile rheumatoid arthritis	555.2	Ulcerative colitis	0.14	0.27	0.3	0.57	0.912
714.2	Juvenile rheumatoid arthritis	709.4	Polymyositis	-0.14	0.27	0.3	0.58	0.912
709.2	Sicca syndrome	555.2	Ulcerative colitis	-0.17	0.33	0.3	0.59	0.912
717	Polymyalgia Rheumatica	695.4	Lupus (localized and systemic)	-0.15	0.28	0.3	0.60	0.912
717	Polymyalgia Rheumatica	714.2	Juvenile rheumatoid arthritis	-0.10	0.21	0.3	0.61	0.912
695.4	Lupus (localized and systemic)	557.1	Celiac disease	-0.21	0.42	0.3	0.61	0.912
571.6	Primary biliary cirrhosis	335	Multiple sclerosis	0.07	0.15	0.3	0.61	0.912
714.2	Juvenile rheumatoid arthritis	362.29	Macular degeneration (senile) of retina NOS	-0.08	0.20	0.2	0.66	0.912
714.1	Rheumatoid arthritis	335	Multiple sclerosis	-0.05	0.13	0.2	0.67	0.912
714.1	Rheumatoid arthritis	709.4	Polymyositis	0.09	0.22	0.2	0.67	0.912
571.6	Primary biliary cirrhosis	362.29	Macular degeneration (senile) of retina NOS	0.07	0.18	0.2	0.67	0.912
714.1	Rheumatoid arthritis	557.1	Celiac disease	-0.10	0.27	0.2	0.69	0.912
714.1	Rheumatoid arthritis	555.2	Ulcerative colitis	0.08	0.22	0.2	0.70	0.912
717	Polymyalgia Rheumatica	555.2	Ulcerative colitis	-0.10	0.27	0.1	0.71	0.912
571.6	Primary biliary cirrhosis	555.2	Ulcerative colitis	-0.08	0.25	0.1	0.72	0.912
717	Polymyalgia Rheumatica	244.4	Hypothyroidism NOS	0.07	0.21	0.1	0.73	0.912
714.2	Juvenile rheumatoid arthritis	557.1	Celiac disease	-0.10	0.32	0.1	0.73	0.912
717	Polymyalgia Rheumatica	715.2	Ankylosing spondylitis	-0.05	0.16	0.1	0.73	0.912
717	Polymyalgia Rheumatica	709.2	Sicca syndrome	-0.09	0.29	0.1	0.74	0.912
717	Polymyalgia Rheumatica	335	Multiple sclerosis	0.05	0.17	0.1	0.75	0.912
715.2	Ankylosing spondylitis	555.2	Ulcerative colitis	-0.05	0.19	0.1	0.77	0.912
715.2	Ankylosing spondylitis	250.1	Type 1 diabetes	-0.02	0.09	0.1	0.78	0.912
715.2	Ankylosing spondylitis	557.1	Celiac disease	-0.07	0.28	0.1	0.80	0.912
695.4	Lupus (localized and systemic)	250.1	Type 1 diabetes	-0.03	0.16	0.1	0.81	0.912
695.4	Lupus (localized and systemic)	244.4	Hypothyroidism NOS	0.05	0.25	0.0	0.82	0.912
709.2	Sicca syndrome	571.6	Primary biliary cirrhosis	0.05	0.25	0.0	0.83	0.912

715.2	Ankylosing spondylitis	335	Multiple sclerosis	-0.03	0.13	0.0	0.83	0.912
557.1	Celiac disease	335	Multiple sclerosis	0.05	0.28	0.0	0.85	0.912
557.1	Celiac disease	555.2	Ulcerative colitis	-0.08	0.42	0.0	0.85	0.912
335	Multiple sclerosis	244.4	Hypothyroidism NOS	0.03	0.15	0.0	0.85	0.912
714.2	Juvenile rheumatoid arthritis	695.4	Lupus (localized and systemic)	-0.05	0.25	0.0	0.85	0.912
715.2	Ankylosing spondylitis	714.1	Rheumatoid arthritis	-0.02	0.13	0.0	0.85	0.912
709.4	Polymyositis	555.2	Ulcerative colitis	0.06	0.35	0.0	0.86	0.912
571.6	Primary biliary cirrhosis	557.1	Celiac disease	0.05	0.32	0.0	0.87	0.912
714.1	Rheumatoid arthritis	362.29	Macular degeneration (senile) of retina NOS	-0.02	0.16	0.0	0.87	0.912
335	Multiple sclerosis	250.1	Type 1 diabetes	0.01	0.10	0.0	0.91	0.935
695.4	Lupus (localized and systemic)	571.6	Primary biliary cirrhosis	-0.03	0.27	0.0	0.92	0.935
717	Polymyalgia Rheumatica	571.6	Primary biliary cirrhosis	-0.02	0.19	0.0	0.92	0.935
715.2	Ankylosing spondylitis	571.6	Primary biliary cirrhosis	0.00	0.14	0.0	1.00	1.000

Supplementary Table 3. Association analyses for imputed HLA alleles and hypothyroidism.

SNP	Ref. allele	Maf cases	MAF controls	OR	95% CI	p-value
HLA-DQA1*01	P	0.37	0.41	0.85	(0.79 - 0.90)	1.4E-07
HLA-DQA1*0501	P	0.26	0.22	1.22	(1.13 - 1.32)	3.1E-07
HLA-DQA1*05	P	0.26	0.22	1.22	(1.13 - 1.32)	3.1E-07
HLA-DRB1*03	P	0.15	0.12	1.31	(1.18 - 1.46)	7.3E-07
HLA-DQB1*0201	P	0.15	0.12	1.30	(1.17 - 1.45)	8.4E-07
HLA-DRB1*0301	P	0.15	0.12	1.31	(1.18 - 1.46)	9.4E-07
HLA-DQA1*0102	P	0.17	0.20	0.83	(0.77 - 0.90)	2.3E-06
HLA-DQB1*06	P	0.22	0.25	0.86	(0.80 - 0.92)	3.0E-05
HLA-C*0501	P	0.11	0.10	1.20	(1.09 - 1.32)	2.7E-04
HLA-C*05	P	0.11	0.10	1.20	(1.09 - 1.32)	2.9E-04
HLA-B*1801	P	0.05	0.04	1.31	(1.13 - 1.51)	3.0E-04
HLA-DRB1*1302	P	0.04	0.05	0.76	(0.65 - 0.88)	3.4E-04
HLA-B*18	P	0.05	0.04	1.31	(1.13 - 1.51)	3.4E-04
HLA-DRB1*1501	P	0.12	0.14	0.86	(0.78 - 0.94)	8.0E-04
HLA-DQB1*02	P	0.24	0.22	1.14	(1.05 - 1.23)	1.3E-03
HLA-DRB1*15	P	0.13	0.15	0.87	(0.80 - 0.95)	2.3E-03
HLA-DQB1*0301	P	0.20	0.18	1.13	(1.04 - 1.22)	2.5E-03
HLA-DRB1*0101	P	0.09	0.10	0.85	(0.77 - 0.95)	2.8E-03
HLA-DQB1*0602	P	0.12	0.13	0.87	(0.80 - 0.96)	3.9E-03
HLA-C*03	P	0.14	0.15	0.88	(0.81 - 0.96)	4.7E-03
HLA-B*1501	P	0.06	0.07	0.84	(0.74 - 0.95)	4.9E-03
HLA-DQB1*0604	P	0.03	0.04	0.79	(0.66 - 0.93)	5.9E-03
HLA-C*0701	P	0.17	0.16	1.14	(1.04 - 1.26)	8.1E-03
HLA-DRB1*13	P	0.10	0.11	0.88	(0.80 - 0.97)	1.1E-02
HLA-B*4402	P	0.11	0.10	1.13	(1.03 - 1.25)	1.3E-02
HLA-DQB1*03	P	0.36	0.35	1.08	(1.02 - 1.16)	1.3E-02
HLA-DQA1*0301	P	0.22	0.20	1.09	(1.01 - 1.17)	2.4E-02
HLA-DQA1*03	P	0.22	0.20	1.09	(1.01 - 1.17)	2.4E-02
HLA-C*12	P	0.05	0.04	1.17	(1.02 - 1.35)	2.6E-02
HLA-C*07	P	0.34	0.32	1.08	(1.01 - 1.16)	2.8E-02
HLA-B*0801	P	0.13	0.12	1.14	(1.01 - 1.29)	3.0E-02
HLA-B*08	P	0.13	0.12	1.14	(1.01 - 1.29)	3.0E-02
HLA-C*0304	P	0.08	0.09	0.89	(0.79 - 0.99)	3.0E-02
HLA-DPB1*05	P	0.02	0.02	0.79	(0.64 - 0.98)	3.5E-02
HLA-DPB1*0501	P	0.02	0.02	0.79	(0.64 - 0.98)	3.5E-02
HLA-DQB1*0501	P	0.11	0.12	0.90	(0.82 - 0.99)	3.5E-02
HLA-DRB1*01	P	0.11	0.12	0.90	(0.82 - 0.99)	3.7E-02
HLA-C*06	P	0.09	0.10	0.90	(0.81 - 0.99)	3.9E-02
HLA-C*0602	P	0.09	0.10	0.90	(0.81 - 0.99)	3.9E-02
HLA-B*2705	P	0.04	0.04	0.85	(0.73 - 0.99)	3.9E-02
HLA-C*1203	P	0.04	0.04	1.18	(1.01 - 1.37)	4.0E-02
HLA-B*15	P	0.07	0.08	0.89	(0.79 - 1.00)	4.2E-02
HLA-DRB1*04	P	0.20	0.19	1.08	(1.00 - 1.17)	4.4E-02
HLA-B*27	P	0.04	0.05	0.86	(0.74 - 1.00)	4.5E-02
HLA-DQB1*05	P	0.15	0.16	0.92	(0.84 - 1.00)	4.8E-02
HLA-DQA1*0101	P	0.13	0.14	0.92	(0.84 - 1.00)	5.2E-02
HLA-DRB1*0401	P	0.12	0.11	1.09	(0.99 - 1.20)	6.8E-02

HLA-DPB1*17	P	0.01	0.01	1.27	(0.98 - 1.65)	7.4E-02
HLA-DPB1*1701	P	0.01	0.01	1.27	(0.98 - 1.65)	7.4E-02
HLA-DQA1*02	P	0.13	0.14	0.93	(0.85 - 1.01)	9.1E-02
HLA-DQA1*0201	P	0.13	0.14	0.93	(0.85 - 1.01)	9.1E-02
HLA-DRB1*0701	P	0.13	0.14	0.93	(0.85 - 1.01)	9.4E-02
HLA-DRB1*07	P	0.13	0.14	0.93	(0.85 - 1.01)	9.4E-02
HLA-B*39	P	0.02	0.01	1.22	(0.96 - 1.54)	1.0E-01
HLA-DRB1*1104	P	0.02	0.02	1.18	(0.96 - 1.45)	1.1E-01
HLA-C*0303	P	0.05	0.06	0.90	(0.79 - 1.03)	1.2E-01
HLA-DRB1*0103	P	0.01	0.01	1.24	(0.94 - 1.63)	1.2E-01
HLA-B*3701	P	0.01	0.02	0.82	(0.64 - 1.06)	1.3E-01
HLA-B*37	P	0.01	0.02	0.82	(0.64 - 1.06)	1.3E-01
HLA-DRB1*11	P	0.09	0.08	1.09	(0.98 - 1.21)	1.3E-01
HLA-B*57	P	0.04	0.04	0.89	(0.76 - 1.04)	1.4E-01
HLA-B*5101	P	0.04	0.05	0.90	(0.77 - 1.04)	1.4E-01
HLA-B*5701	P	0.04	0.04	0.89	(0.76 - 1.04)	1.4E-01
HLA-B*1302	P	0.02	0.02	0.86	(0.70 - 1.06)	1.5E-01
HLA-B*44	P	0.16	0.15	1.06	(0.98 - 1.16)	1.6E-01
HLA-DRB1*0404	P	0.05	0.05	1.10	(0.96 - 1.26)	1.6E-01
HLA-DQA1*0401	P	0.03	0.03	1.13	(0.95 - 1.35)	1.6E-01
HLA-DQA1*04	P	0.03	0.03	1.13	(0.95 - 1.35)	1.6E-01
HLA-DPA1*01	A	0.17	0.18	0.95	(0.87 - 1.02)	1.6E-01
HLA-DPA1*02	P	0.17	0.18	0.95	(0.87 - 1.02)	1.6E-01
HLA-B*13	P	0.02	0.02	0.87	(0.71 - 1.06)	1.7E-01
HLA-DRB1*09	P	0.01	0.01	1.20	(0.92 - 1.55)	1.7E-01
HLA-DRB1*0901	P	0.01	0.01	1.20	(0.92 - 1.55)	1.7E-01
HLA-B*51	P	0.04	0.05	0.91	(0.78 - 1.05)	1.9E-01
HLA-DQB1*0303	P	0.05	0.05	0.91	(0.79 - 1.05)	1.9E-01
HLA-DPB1*04	A	0.45	0.46	0.96	(0.91 - 1.02)	1.9E-01
HLA-A*2402	P	0.08	0.08	0.93	(0.84 - 1.04)	1.9E-01
HLA-B*3503	P	0.02	0.01	1.17	(0.92 - 1.49)	2.0E-01
HLA-A*24	P	0.08	0.09	0.93	(0.84 - 1.04)	2.0E-01
HLA-DPA1*0202	P	0.03	0.03	0.90	(0.75 - 1.07)	2.2E-01
HLA-B*4403	P	0.05	0.05	0.92	(0.80 - 1.05)	2.2E-01
HLA-DPA1*0103	A	0.18	0.18	0.95	(0.88 - 1.03)	2.3E-01
HLA-A*2301	P	0.02	0.01	1.16	(0.91 - 1.47)	2.3E-01
HLA-A*23	P	0.02	0.01	1.16	(0.91 - 1.47)	2.3E-01
HLA-A*3001	P	0.01	0.01	0.85	(0.64 - 1.12)	2.5E-01
HLA-A*03	P	0.15	0.14	1.05	(0.96 - 1.15)	2.6E-01
HLA-DRB1*08	P	0.03	0.03	1.10	(0.93 - 1.30)	2.7E-01
HLA-DRB1*1101	P	0.05	0.05	1.08	(0.94 - 1.23)	2.8E-01
HLA-DPB1*0301	P	0.11	0.11	0.95	(0.86 - 1.04)	2.8E-01
HLA-DPB1*03	P	0.11	0.11	0.95	(0.86 - 1.04)	2.8E-01
HLA-C*1601	P	0.03	0.04	0.91	(0.78 - 1.08)	2.8E-01
HLA-A*0301	P	0.14	0.14	1.05	(0.96 - 1.14)	2.9E-01
HLA-A*02	P	0.30	0.30	1.04	(0.97 - 1.11)	2.9E-01
HLA-A*3101	P	0.03	0.03	0.91	(0.75 - 1.09)	3.0E-01
HLA-A*3201	P	0.03	0.03	0.92	(0.77 - 1.08)	3.0E-01
HLA-A*32	P	0.03	0.03	0.92	(0.77 - 1.08)	3.0E-01
HLA-B*1401	P	0.01	0.01	0.85	(0.63 - 1.16)	3.0E-01
HLA-B*35	P	0.08	0.08	1.06	(0.95 - 1.18)	3.1E-01
HLA-B*4001	P	0.06	0.07	0.94	(0.83 - 1.06)	3.1E-01

HLA-C*16	P	0.04	0.04	0.92	(0.79 - 1.08)	3.2E-01
HLA-DRB1*0801	P	0.02	0.02	1.11	(0.91 - 1.36)	3.2E-01
HLA-C*15	P	0.02	0.02	0.90	(0.74 - 1.11)	3.2E-01
HLA-A*31	P	0.03	0.03	0.91	(0.76 - 1.10)	3.3E-01
HLA-B*14	P	0.04	0.04	0.92	(0.79 - 1.09)	3.4E-01
HLA-DQB1*04	P	0.03	0.03	1.09	(0.91 - 1.31)	3.5E-01
HLA-DPB1*0401	P	0.44	0.43	1.03	(0.97 - 1.09)	3.5E-01
HLA-DQB1*0402	P	0.03	0.03	1.09	(0.91 - 1.31)	3.5E-01
HLA-DPA1*0201	P	0.14	0.14	0.96	(0.88 - 1.05)	3.7E-01
HLA-DRB1*1201	P	0.01	0.01	1.13	(0.87 - 1.46)	3.7E-01
HLA-DRB1*12	P	0.01	0.01	1.13	(0.87 - 1.46)	3.7E-01
HLA-C*1402	P	0.01	0.01	0.88	(0.66 - 1.17)	3.7E-01
HLA-C*14	P	0.01	0.01	0.88	(0.66 - 1.17)	3.7E-01
HLA-B*3901	P	0.01	0.01	1.14	(0.85 - 1.54)	3.8E-01
HLA-DQB1*0302	P	0.12	0.11	1.04	(0.95 - 1.15)	3.8E-01
HLA-A*0201	P	0.29	0.29	1.03	(0.96 - 1.10)	4.0E-01
HLA-DQB1*0603	P	0.06	0.06	0.95	(0.84 - 1.07)	4.0E-01
HLA-DPB1*1101	P	0.02	0.02	0.92	(0.75 - 1.12)	4.0E-01
HLA-DPB1*11	P	0.02	0.02	0.92	(0.75 - 1.12)	4.0E-01
HLA-C*0202	P	0.04	0.04	0.94	(0.80 - 1.09)	4.1E-01
HLA-C*02	P	0.04	0.04	0.94	(0.80 - 1.09)	4.1E-01
HLA-DPB1*1301	P	0.02	0.02	0.91	(0.72 - 1.15)	4.2E-01
HLA-DPB1*13	P	0.02	0.02	0.91	(0.72 - 1.15)	4.2E-01
HLA-B*40	P	0.07	0.08	0.96	(0.85 - 1.07)	4.3E-01
HLA-C*1502	P	0.02	0.02	0.92	(0.75 - 1.14)	4.7E-01
HLA-A*30	P	0.02	0.02	1.07	(0.88 - 1.31)	4.9E-01
HLA-A*68	P	0.04	0.04	0.95	(0.81 - 1.10)	4.9E-01
HLA-A*6801	P	0.03	0.03	0.94	(0.80 - 1.12)	5.0E-01
HLA-DPB1*0402	P	0.11	0.11	1.03	(0.94 - 1.13)	5.2E-01
HLA-C*08	P	0.04	0.04	0.95	(0.81 - 1.12)	5.3E-01
HLA-DPB1*1001	P	0.02	0.02	0.93	(0.74 - 1.17)	5.5E-01
HLA-DPB1*10	P	0.02	0.02	0.93	(0.74 - 1.17)	5.5E-01
HLA-DPB1*02	P	0.14	0.13	1.03	(0.94 - 1.12)	5.5E-01
HLA-C*0802	P	0.04	0.04	0.95	(0.81 - 1.12)	5.6E-01
HLA-B*4002	P	0.01	0.01	1.09	(0.81 - 1.46)	5.7E-01
HLA-A*2902	P	0.04	0.04	1.04	(0.89 - 1.22)	5.9E-01
HLA-DPB1*0601	P	0.02	0.02	0.95	(0.78 - 1.16)	6.0E-01
HLA-DPB1*06	P	0.02	0.02	0.95	(0.78 - 1.16)	6.0E-01
HLA-A*2501	P	0.02	0.02	0.94	(0.76 - 1.17)	6.0E-01
HLA-A*25	P	0.02	0.02	0.94	(0.76 - 1.17)	6.0E-01
HLA-A*29	P	0.04	0.04	1.04	(0.89 - 1.21)	6.2E-01
HLA-DRB1*1301	P	0.06	0.06	0.97	(0.85 - 1.10)	6.3E-01
HLA-A*1101	P	0.07	0.07	0.97	(0.86 - 1.10)	6.3E-01
HLA-DQB1*0202	P	0.09	0.10	0.98	(0.88 - 1.08)	6.3E-01
HLA-B*1402	P	0.03	0.03	0.96	(0.79 - 1.15)	6.4E-01
HLA-C*0401	P	0.09	0.09	0.98	(0.88 - 1.08)	6.7E-01
HLA-A*11	P	0.07	0.07	0.97	(0.86 - 1.10)	6.7E-01
HLA-C*04	P	0.09	0.09	0.98	(0.88 - 1.09)	6.9E-01
HLA-DPB1*1401	P	0.01	0.01	0.95	(0.72 - 1.25)	7.0E-01
HLA-DPB1*14	P	0.01	0.01	0.95	(0.72 - 1.25)	7.0E-01
HLA-A*0101	P	0.17	0.16	0.98	(0.88 - 1.09)	7.1E-01
HLA-DPB1*0101	P	0.06	0.06	1.03	(0.90 - 1.17)	7.2E-01

HLA-DPB1*01	P	0.06	0.06	1.03	(0.90 - 1.17)	7.2E-01
HLA-C*0102	P	0.03	0.03	0.98	(0.82 - 1.16)	7.8E-01
HLA-C*01	P	0.03	0.03	0.98	(0.82 - 1.16)	7.8E-01
HLA-DQB1*0503	P	0.02	0.03	0.97	(0.81 - 1.18)	7.9E-01
HLA-C*0702	P	0.15	0.15	1.01	(0.93 - 1.10)	7.9E-01
HLA-B*38	P	0.01	0.01	0.97	(0.74 - 1.28)	8.3E-01
HLA-B*3801	P	0.01	0.01	0.97	(0.74 - 1.28)	8.3E-01
HLA-B*07	P	0.14	0.14	0.99	(0.91 - 1.08)	8.6E-01
HLA-DRB1*0407	P	0.01	0.01	1.02	(0.78 - 1.34)	8.6E-01
HLA-B*4901	P	0.01	0.02	0.98	(0.76 - 1.25)	8.7E-01
HLA-B*49	P	0.01	0.02	0.98	(0.76 - 1.25)	8.7E-01
HLA-DQB1*0502	P	0.01	0.01	1.02	(0.77 - 1.36)	8.8E-01
HLA-DPB1*0201	P	0.13	0.13	1.00	(0.91 - 1.09)	9.2E-01
HLA-DQA1*0103	P	0.06	0.06	0.99	(0.88 - 1.12)	9.3E-01
HLA-B*3501	P	0.05	0.05	1.00	(0.87 - 1.14)	9.4E-01
HLA-B*0702	P	0.14	0.14	1.00	(0.91 - 1.09)	9.6E-01
HLA-DRB1*14	P	0.02	0.02	1.01	(0.82 - 1.23)	9.6E-01
HLA-C*0704	P	0.02	0.02	1.01	(0.80 - 1.26)	9.6E-01
HLA-A*2601	P	0.02	0.02	1.00	(0.82 - 1.22)	9.7E-01
HLA-A*26	P	0.02	0.02	1.00	(0.82 - 1.21)	9.8E-01
HLA-B*5501	P	0.02	0.02	1.00	(0.82 - 1.23)	9.8E-01
HLA-B*55	P	0.02	0.02	1.00	(0.82 - 1.23)	9.8E-01
HLA-DRB1*1401	P	0.02	0.02	1.00	(0.79 - 1.26)	9.8E-01

Supplementary Table4. Association analyses for imputed HLA alleles and polymyalgia rheumatica.

SNP	Ref. allele	Maf cases	MAF controls	OR	95% CI	p-value
HLA-DQA1*0301	P	0.28	0.20	1.56	(1.33 - 1.83)	7.0E-08
HLA-DQA1*03	P	0.28	0.20	1.56	(1.33 - 1.83)	7.0E-08
HLA-DRB1*04	P	0.26	0.19	1.55	(1.32 - 1.83)	2.0E-07
HLA-DRB1*0401	P	0.16	0.11	1.57	(1.29 - 1.92)	1.1E-05
HLA-DQA1*0201	P	0.08	0.14	0.59	(0.46 - 0.76)	4.2E-05
HLA-DQA1*02	P	0.08	0.14	0.59	(0.46 - 0.76)	4.2E-05
HLA-DRB1*0701	P	0.08	0.14	0.59	(0.46 - 0.76)	4.2E-05
HLA-DRB1*07	P	0.08	0.14	0.59	(0.46 - 0.76)	4.2E-05
HLA-DQB1*0202	P	0.06	0.10	0.56	(0.42 - 0.76)	1.7E-04
HLA-C*06	P	0.05	0.09	0.56	(0.41 - 0.77)	2.6E-04
HLA-C*0602	P	0.05	0.09	0.56	(0.41 - 0.77)	2.6E-04
HLA-DQB1*02	P	0.19	0.23	0.70	(0.57 - 0.86)	4.7E-04
HLA-DQB1*0302	P	0.15	0.11	1.44	(1.17 - 1.77)	4.7E-04
HLA-DQB1*03	P	0.40	0.35	1.28	(1.11 - 1.49)	8.6E-04
HLA-DQB1*0604	P	0.02	0.03	0.50	(0.29 - 0.85)	1.1E-02
HLA-C*1601	P	0.02	0.04	0.56	(0.35 - 0.89)	1.3E-02
HLA-DRB1*1302	P	0.03	0.04	0.58	(0.37 - 0.90)	1.5E-02
HLA-A*2902	P	0.02	0.04	0.56	(0.35 - 0.90)	1.6E-02
HLA-DPB1*1401	P	0.02	0.01	1.73	(1.08 - 2.77)	2.2E-02
HLA-DPB1*14	P	0.02	0.01	1.73	(1.08 - 2.77)	2.2E-02
HLA-C*16	P	0.03	0.05	0.61	(0.40 - 0.93)	2.2E-02
HLA-B*13	P	0.01	0.02	0.44	(0.22 - 0.89)	2.3E-02
HLA-B*1302	P	0.01	0.02	0.44	(0.22 - 0.90)	2.4E-02
HLA-DRB1*0404	P	0.06	0.05	1.40	(1.04 - 1.88)	2.5E-02
HLA-DQB1*0503	P	0.01	0.03	0.50	(0.27 - 0.92)	2.6E-02
HLA-B*49	P	0.02	0.01	1.66	(1.03 - 2.67)	3.7E-02
HLA-B*4901	P	0.02	0.01	1.66	(1.03 - 2.67)	3.7E-02
HLA-B*07	P	0.16	0.14	1.23	(1.01 - 1.48)	3.7E-02
HLA-C*0102	P	0.05	0.03	1.42	(1.02 - 1.98)	3.9E-02
HLA-C*01	P	0.05	0.03	1.42	(1.02 - 1.98)	3.9E-02
HLA-DRB1*14	P	0.01	0.02	0.52	(0.28 - 0.98)	4.5E-02
HLA-B*0702	P	0.16	0.14	1.21	(1.00 - 1.47)	5.3E-02
HLA-A*29	P	0.03	0.04	0.66	(0.43 - 1.01)	5.5E-02
HLA-DPB1*1701	P	0.02	0.01	1.74	(0.99 - 3.06)	5.6E-02
HLA-DPB1*17	P	0.02	0.01	1.74	(0.99 - 3.06)	5.6E-02
HLA-DQB1*0301	P	0.21	0.18	1.18	(0.99 - 1.41)	6.8E-02
HLA-B*4403	P	0.04	0.05	0.71	(0.49 - 1.03)	6.9E-02
HLA-C*08	P	0.04	0.03	1.38	(0.97 - 1.96)	7.3E-02
HLA-DRB1*1401	P	0.01	0.02	0.50	(0.23 - 1.07)	7.4E-02
HLA-C*0802	P	0.04	0.03	1.36	(0.95 - 1.94)	9.2E-02
HLA-B*5701	P	0.03	0.04	0.69	(0.45 - 1.06)	9.2E-02
HLA-A*3101	P	0.04	0.03	1.38	(0.94 - 2.03)	9.7E-02

HLA-A*31	P	0.04	0.03	1.38	(0.94 - 2.02)	1.0E-01
HLA-C*0704	P	0.01	0.02	0.57	(0.29 - 1.12)	1.0E-01
HLA-DRB1*1501	P	0.16	0.14	1.17	(0.96 - 1.43)	1.1E-01
HLA-A*0101	P	0.16	0.17	0.82	(0.64 - 1.05)	1.2E-01
HLA-C*0702	P	0.17	0.15	1.16	(0.96 - 1.41)	1.2E-01
HLA-B*57	P	0.03	0.04	0.71	(0.46 - 1.09)	1.2E-01
HLA-DPB1*03	P	0.10	0.11	0.83	(0.65 - 1.05)	1.2E-01
HLA-DPB1*0301	P	0.10	0.11	0.83	(0.65 - 1.05)	1.2E-01
HLA-A*3001	P	0.01	0.01	0.49	(0.20 - 1.20)	1.2E-01
HLA-DPB1*04	A	0.43	0.46	0.90	(0.78 - 1.04)	1.4E-01
HLA-DRB1*0801	P	0.01	0.02	0.64	(0.35 - 1.17)	1.5E-01
HLA-B*37	P	0.01	0.01	0.57	(0.27 - 1.22)	1.5E-01
HLA-B*3701	P	0.01	0.01	0.57	(0.27 - 1.22)	1.5E-01
HLA-B*5101	P	0.04	0.05	0.77	(0.53 - 1.11)	1.6E-01
HLA-DRB1*1201	P	0.01	0.01	0.56	(0.24 - 1.27)	1.6E-01
HLA-DRB1*12	P	0.01	0.01	0.56	(0.24 - 1.27)	1.6E-01
HLA-DQB1*0602	P	0.15	0.13	1.15	(0.94 - 1.40)	1.8E-01
HLA-B*14	P	0.04	0.03	1.28	(0.89 - 1.83)	1.8E-01
HLA-DPB1*10	P	0.02	0.02	1.38	(0.86 - 2.22)	1.8E-01
HLA-DPB1*1001	P	0.02	0.02	1.38	(0.86 - 2.22)	1.8E-01
HLA-DRB1*15	P	0.16	0.14	1.14	(0.94 - 1.39)	1.8E-01
HLA-DRB1*0407	P	0.02	0.01	1.44	(0.84 - 2.47)	1.9E-01
HLA-DPB1*0601	P	0.03	0.02	1.32	(0.84 - 2.06)	2.2E-01
HLA-DPB1*06	P	0.03	0.02	1.32	(0.84 - 2.06)	2.2E-01
HLA-DQA1*04	P	0.02	0.03	0.74	(0.45 - 1.21)	2.3E-01
HLA-DQA1*0401	P	0.02	0.03	0.74	(0.45 - 1.21)	2.3E-01
HLA-DQA1*0501	P	0.22	0.23	0.89	(0.74 - 1.08)	2.3E-01
HLA-DQA1*05	P	0.22	0.23	0.89	(0.74 - 1.08)	2.3E-01
HLA-DPB1*1101	P	0.02	0.02	0.73	(0.43 - 1.24)	2.4E-01
HLA-DPB1*11	P	0.02	0.02	0.73	(0.43 - 1.24)	2.4E-01
HLA-DRB1*09	P	0.02	0.01	1.37	(0.81 - 2.33)	2.5E-01
HLA-DRB1*0901	P	0.02	0.01	1.37	(0.81 - 2.33)	2.5E-01
HLA-DPB1*0401	P	0.45	0.43	1.09	(0.94 - 1.25)	2.5E-01
HLA-B*51	P	0.04	0.05	0.81	(0.57 - 1.16)	2.5E-01
HLA-DQB1*0501	P	0.13	0.12	1.13	(0.91 - 1.40)	2.6E-01
HLA-C*07	P	0.35	0.33	1.10	(0.93 - 1.30)	2.6E-01
HLA-DRB1*1101	P	0.04	0.05	0.82	(0.58 - 1.17)	2.7E-01
HLA-A*26	P	0.02	0.02	0.75	(0.44 - 1.26)	2.7E-01
HLA-DRB1*08	P	0.02	0.03	0.77	(0.49 - 1.23)	2.8E-01
HLA-DRB1*13	P	0.10	0.11	0.88	(0.69 - 1.11)	2.8E-01
HLA-A*2601	P	0.02	0.02	0.75	(0.44 - 1.27)	2.8E-01
HLA-C*1203	P	0.04	0.04	1.20	(0.84 - 1.70)	3.1E-01
HLA-B*1402	P	0.03	0.02	1.25	(0.80 - 1.93)	3.3E-01
HLA-DRB1*0101	P	0.11	0.10	1.12	(0.89 - 1.41)	3.3E-01
HLA-C*0501	P	0.11	0.10	1.12	(0.89 - 1.40)	3.5E-01
HLA-A*3201	P	0.04	0.03	1.20	(0.82 - 1.75)	3.5E-01
HLA-A*32	P	0.04	0.03	1.20	(0.82 - 1.75)	3.5E-01

HLA-C*05	P	0.11	0.10	1.12	(0.89 - 1.40)	3.5E-01
HLA-B*1401	P	0.01	0.01	1.33	(0.73 - 2.43)	3.5E-01
HLA-DQB1*0303	P	0.04	0.05	0.85	(0.61 - 1.20)	3.7E-01
HLA-C*1402	P	0.01	0.01	0.73	(0.35 - 1.49)	3.8E-01
HLA-C*14	P	0.01	0.01	0.73	(0.35 - 1.49)	3.8E-01
HLA-DRB1*01	P	0.13	0.12	1.10	(0.89 - 1.36)	3.8E-01
HLA-DRB1*0301	P	0.13	0.13	0.89	(0.69 - 1.16)	4.0E-01
HLA-DQB1*0201	P	0.13	0.13	0.90	(0.69 - 1.16)	4.1E-01
HLA-B*4402	P	0.11	0.10	1.10	(0.87 - 1.38)	4.2E-01
HLA-A*2501	P	0.02	0.02	1.22	(0.75 - 1.99)	4.3E-01
HLA-A*25	P	0.02	0.02	1.22	(0.75 - 1.99)	4.3E-01
HLA-DPB1*02	P	0.12	0.13	0.92	(0.74 - 1.14)	4.3E-01
HLA-A*30	P	0.02	0.02	0.82	(0.49 - 1.36)	4.4E-01
HLA-DQB1*0502	P	0.01	0.01	0.74	(0.35 - 1.59)	4.4E-01
HLA-B*39	P	0.01	0.02	0.78	(0.41 - 1.50)	4.6E-01
HLA-DRB1*03	P	0.13	0.13	0.91	(0.70 - 1.18)	4.7E-01
HLA-C*1502	P	0.02	0.02	0.82	(0.48 - 1.41)	4.7E-01
HLA-A*68	P	0.04	0.04	1.14	(0.79 - 1.62)	4.9E-01
HLA-A*6801	P	0.04	0.03	1.15	(0.78 - 1.69)	4.9E-01
HLA-B*15	P	0.07	0.08	0.91	(0.69 - 1.19)	5.0E-01
HLA-B*4002	P	0.01	0.01	0.75	(0.33 - 1.72)	5.0E-01
HLA-DPA1*0201	P	0.15	0.14	1.07	(0.87 - 1.30)	5.2E-01
HLA-DRB1*11	P	0.08	0.08	0.92	(0.70 - 1.20)	5.3E-01
HLA-C*15	P	0.02	0.02	0.85	(0.51 - 1.42)	5.4E-01
HLA-DPB1*0402	P	0.12	0.11	1.07	(0.86 - 1.33)	5.4E-01
HLA-DPB1*0201	P	0.12	0.13	0.94	(0.75 - 1.16)	5.5E-01
HLA-B*3801	P	0.01	0.01	1.18	(0.65 - 2.16)	5.8E-01
HLA-A*0301	P	0.13	0.14	0.94	(0.76 - 1.17)	5.9E-01
HLA-DPB1*1301	P	0.02	0.02	1.15	(0.69 - 1.93)	5.9E-01
HLA-DPB1*13	P	0.02	0.02	1.15	(0.69 - 1.93)	5.9E-01
HLA-DQB1*0402	P	0.02	0.02	0.88	(0.54 - 1.42)	5.9E-01
HLA-B*3901	P	0.01	0.01	0.81	(0.38 - 1.75)	5.9E-01
HLA-B*38	P	0.01	0.01	1.18	(0.65 - 2.14)	6.0E-01
HLA-A*1101	P	0.07	0.07	1.08	(0.81 - 1.43)	6.0E-01
HLA-A*11	P	0.07	0.07	1.08	(0.81 - 1.43)	6.0E-01
HLA-DPA1*0202	P	0.03	0.03	0.90	(0.59 - 1.38)	6.3E-01
HLA-C*12	P	0.05	0.04	1.08	(0.77 - 1.52)	6.5E-01
HLA-C*0701	P	0.17	0.16	1.05	(0.84 - 1.32)	6.6E-01
HLA-C*0202	P	0.04	0.04	0.92	(0.64 - 1.33)	6.7E-01
HLA-C*02	P	0.04	0.04	0.92	(0.64 - 1.33)	6.7E-01
HLA-B*44	P	0.15	0.16	0.96	(0.78 - 1.17)	6.8E-01
HLA-DQB1*04	P	0.02	0.02	0.91	(0.57 - 1.46)	7.0E-01
HLA-A*02	P	0.29	0.30	0.97	(0.83 - 1.14)	7.1E-01
HLA-DPA1*02	P	0.18	0.17	1.04	(0.86 - 1.25)	7.1E-01
HLA-DQA1*01	P	0.39	0.40	0.97	(0.84 - 1.13)	7.1E-01
HLA-C*0303	P	0.06	0.06	0.95	(0.70 - 1.28)	7.2E-01
HLA-DQB1*05	P	0.15	0.16	0.97	(0.79 - 1.18)	7.3E-01

HLA-B*4001	P	0.06	0.06	1.05	(0.78 - 1.42)	7.3E-01
HLA-DQB1*0603	P	0.07	0.06	1.05	(0.79 - 1.40)	7.3E-01
HLA-DPA1*01	A	0.18	0.17	1.03	(0.86 - 1.24)	7.4E-01
HLA-A*03	P	0.14	0.15	0.97	(0.78 - 1.19)	7.4E-01
HLA-B*2705	P	0.05	0.04	1.06	(0.76 - 1.48)	7.4E-01
HLA-DQA1*0102	P	0.19	0.20	0.97	(0.81 - 1.17)	7.6E-01
HLA-DPB1*0101	P	0.06	0.06	0.95	(0.69 - 1.31)	7.7E-01
HLA-DPB1*01	P	0.06	0.06	0.95	(0.69 - 1.31)	7.7E-01
HLA-DRB1*1301	P	0.06	0.06	1.05	(0.77 - 1.41)	7.7E-01
HLA-B*27	P	0.05	0.05	1.05	(0.75 - 1.46)	7.8E-01
HLA-B*1501	P	0.07	0.07	0.96	(0.72 - 1.28)	8.0E-01
HLA-DPB1*0501	P	0.02	0.02	1.06	(0.64 - 1.74)	8.2E-01
HLA-DPB1*05	P	0.02	0.02	1.06	(0.64 - 1.74)	8.2E-01
HLA-B*5501	P	0.02	0.02	0.95	(0.58 - 1.57)	8.4E-01
HLA-B*55	P	0.02	0.02	0.95	(0.58 - 1.57)	8.4E-01
HLA-B*35	P	0.08	0.08	0.97	(0.75 - 1.27)	8.5E-01
HLA-B*1801	P	0.04	0.04	0.97	(0.67 - 1.39)	8.6E-01
HLA-B*18	P	0.04	0.04	0.97	(0.67 - 1.39)	8.6E-01
HLA-A*2402	P	0.08	0.08	1.02	(0.79 - 1.32)	8.7E-01
HLA-B*3501	P	0.06	0.06	1.03	(0.75 - 1.40)	8.7E-01
HLA-B*3503	P	0.01	0.01	1.05	(0.58 - 1.91)	8.8E-01
HLA-A*2301	P	0.02	0.02	1.04	(0.61 - 1.79)	8.8E-01
HLA-A*23	P	0.02	0.02	1.04	(0.61 - 1.79)	8.8E-01
HLA-C*0304	P	0.08	0.08	1.02	(0.79 - 1.33)	8.8E-01
HLA-DQA1*0101	P	0.14	0.14	0.99	(0.80 - 1.21)	8.9E-01
HLA-A*24	P	0.08	0.08	1.02	(0.79 - 1.31)	8.9E-01
HLA-DRB1*1104	P	0.02	0.02	0.97	(0.60 - 1.57)	9.0E-01
HLA-DPA1*0103	A	0.19	0.18	1.01	(0.84 - 1.21)	9.2E-01
HLA-DQB1*06	P	0.24	0.25	0.99	(0.84 - 1.17)	9.3E-01
HLA-B*40	P	0.07	0.07	1.01	(0.77 - 1.34)	9.3E-01
HLA-DRB1*0103	P	0.01	0.01	1.03	(0.53 - 1.97)	9.4E-01
HLA-C*04	P	0.09	0.10	0.99	(0.78 - 1.27)	9.5E-01
HLA-DQA1*0103	P	0.06	0.06	0.99	(0.74 - 1.33)	9.6E-01
HLA-A*0201	P	0.29	0.29	1.00	(0.86 - 1.18)	9.6E-01
HLA-C*0401	P	0.09	0.09	0.99	(0.78 - 1.27)	9.7E-01
HLA-B*0801	P	0.13	0.12	0.99	(0.74 - 1.33)	9.7E-01
HLA-B*08	P	0.13	0.12	0.99	(0.74 - 1.33)	9.7E-01
HLA-C*03	P	0.14	0.15	1.00	(0.81 - 1.22)	9.7E-01
